# Negative chemotaxis of *Ligilactobacillus agilis* BKN88 against gut-derived substances

**DOI:** 10.1038/s41598-023-42840-5

**Published:** 2023-09-20

**Authors:** Shunya Suzuki, Kenji Yokota, Shizunobu Igimi, Akinobu Kajikawa

**Affiliations:** 1 Department of Agricultural Chemistry, Graduate School of Tokyo University of Agriculture, 1-1-1 Sakuragaoka, Setagaya, Tokyo 156-8502 Japan; 2 Bioproduction Research Institute, National Institute of Advanced Industrial Science and Technology, 1-1-1 Higashi, Tsukuba, Ibaraki 305-8566 Japan

**Keywords:** Microbiology, Bacteria, Bacteriology, Cellular microbiology, Microbial communities, Microbial genetics

## Abstract

*Ligilactobacillus agilis* is a motile lactic acid bacterium found in the gastrointestinal tracts of animals. The findings of our previous study suggest that the motility of *L. agilis* BKN88 enables gut colonization in murine models. However, the chemotactic abilities of motile lactobacilli remain unknown. This study aimed to identify the gut-derived chemoeffectors and their corresponding chemoreceptors in *L. agilis* BKN88. Chemotaxis assays with chemotactic and non-chemotactic (Δ*cheA*) *L. agilis* strains revealed that low pH, organic acids, and bile salts served as repellents. *L. agilis *BKN88 was more sensitive to bile and acid than the gut-derived non-motile lactobacilli, implying that *L. agilis* might utilize motility and chemotaxis instead of exhibiting stress tolerance/resistance. *L. agilis *BKN88 contains five putative chemoreceptor genes (*mcp1*–*mcp5*). Chemotaxis assays using a series of chemoreceptor mutants revealed that each of the five chemoreceptors could sense multiple chemoeffectors and that these chemoreceptors were functionally redundant. Mcp2 and Mcp3 sensed all tested chemoeffectors. This study provides further insights into the interactions between chemoreceptors and ligands of motile lactobacilli and the unique ecological and evolutionary features of motile lactobacilli, which may be distinct from those of non-motile lactobacilli.

## Introduction

Bacterial chemotaxis is the movement of motile bacteria toward favorable chemicals or away from unfavorable chemicals and is mediated by chemotactic signaling pathways^1–3^. The molecular mechanisms underlying chemotactic signaling have been intensely studied in *Escherichia coli* and *Salmonella enterica* serovar *Typhimurium*^4,5^. Chemotaxis is triggered by the binding of ligands (chemoeffectors) to chemoreceptors called methyl-accepting chemotaxis proteins (MCPs) and transducer-like proteins (Tlps). Subsequently, chemoreceptors transduce signals through a series of cytoplasmic chemotactic proteins and modulate flagellar motor rotation^6,7^. This chemotactic signaling pathway is relatively conserved across bacteria, whereas chemoeffectors and chemoreceptors vary among bacterial species/strains.

In the last few decades, several chemoeffectors have been identified, and various chemoreceptors have been functionally characterized. For example, urea^8–11^, lactate^12^, various amino acids^8,13^, and mucin^14^ serve as chemoattractants for *Helicobacter pylori*, whereas low pH^11,15,16^, reactive oxygen species^17^ and autoinducer-2^18^ serve as chemorepellents. Four chemoreceptors (TlpA, TlpB, TlpC, and TlpD) are mediated by these chemotactic responses, which are required to colonize the gastric mucosa efficiently^19,20^. Ten chemoreceptors have been identified in the gram-positive model bacterium *Bacillus subtilis*, 10 chemoreceptors have been found, and some have been characterized. McpB and McpC mediate chemotaxis toward amino acids^21–23^, whereas McpA mediates chemoattraction toward glucose and α-methylglucoside^22^. Although most studies have examined the chemoeffectors and chemoreceptors of pathogenic bacteria and some bacterial species^6,24^, limited studies have focused on beneficial gut microbes such as lactic acid bacteria.

Some lactobacilli, considered beneficial commensals, are found in the gastrointestinal tract of humans and animals. Most lactobacilli are non-motile and do not possess motility genes, although a small proportion of lactobacilli are motile^25,26^. The ecology of non-motile lactobacilli has been relatively well studied^27–30^, whereas that of motile lactobacilli is poorly characterized. Previously, we demonstrated that *Ligilactobacillus agilis* (previously named *Lactobacillus agilis*) BKN88, a motile strain isolated from chicken, utilizes motility for gut colonization in murine models^31^. Thus, the chemotaxis of motile lactobacilli may be involved in gut colonization. However, this has not been demonstrated owing to a lack of information on the chemoeffectors and chemoreceptors of motile lactobacilli. This study aimed to identify gut-derived chemoeffectors and their corresponding chemoreceptors in *L. agilis* BKN88 to gain insights into its ecology and evolution.

## Results

*L. agilis* BKN88 has only one histidine kinase (CheA) that plays a central role in chemotactic signaling. Thus, a motile but non-chemotactic derivative of *L. agilis* BKN88 was constructed by deleting *cheA* and was used as a negative control in the chemotaxis assay. Deletion of *cheA* was confirmed by polymerase chain reaction (PCR). PCR analysis of the *cheA* deletion mutant (Δ*cheA*) revealed an amplicon with the expected size (Supplementary Fig. S1a, b). The swimming behavior of the Δ*cheA* mutant was examined using optical microscopy. As shown in Supplementary Fig. S1c, the Δ*cheA* mutant exhibited motility with a higher frequency of tumbling than the wild-type (WT) strain, similar to *B. subtilis*^32^. Preliminary analysis of the chemotaxis of chemotactic and non-chemotactic (Δ*cheA*) *L. agilis *strains was performed using the capillary and microscopic agar-drop assays^31,33^. Among these assays, the microscopic agar-drop assay was chosen for further studies on chemotaxis in *L. agilis* owing to its high reproducibility (Supplementary Fig. S2).

### Chemotactic responses of *L. agilis* to pH

**Figure 1:**
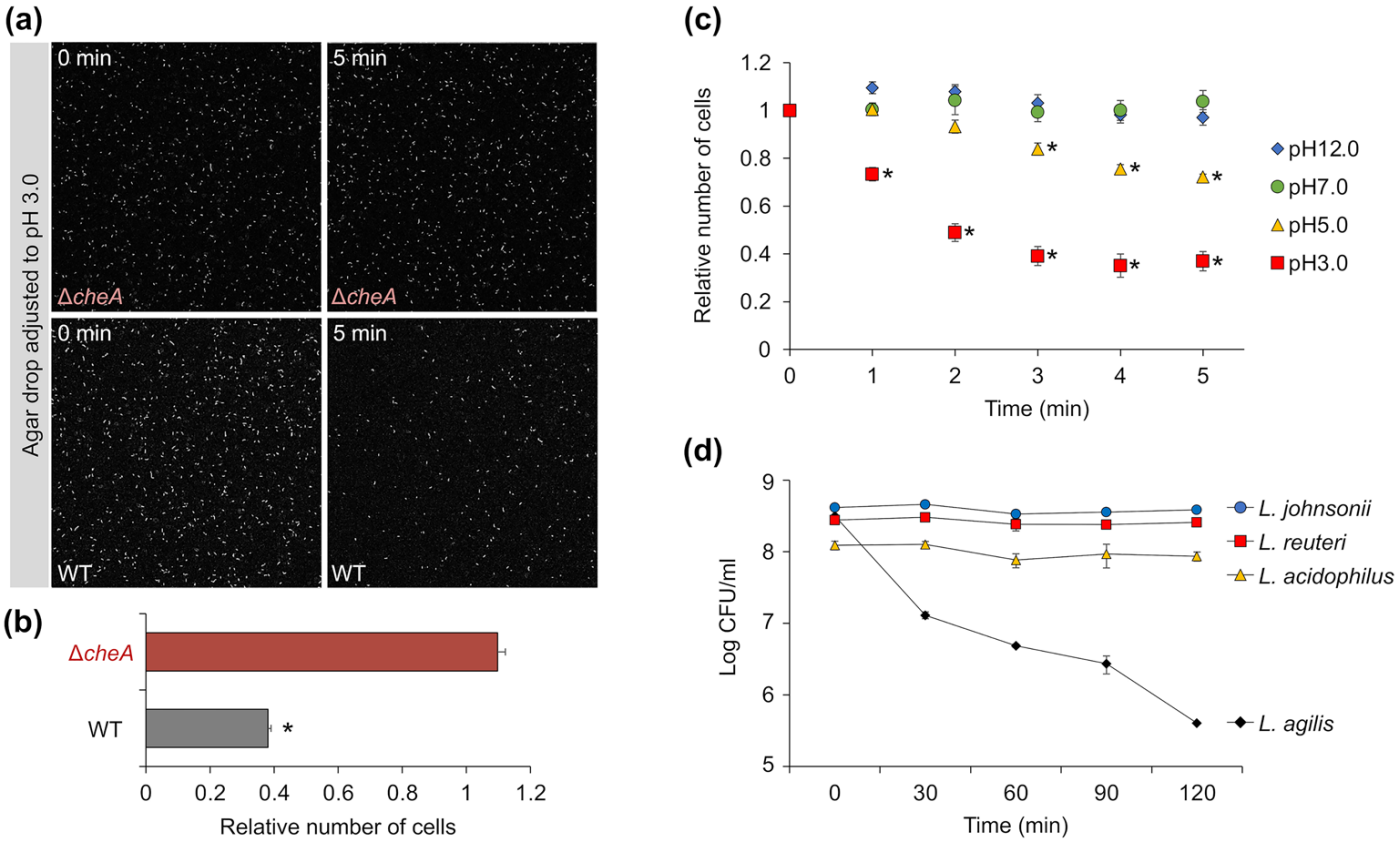
Chemotactic responses of *Ligilactobacillus agilis* to pH. (**a**, **b**) Chemotaxis toward acidic pH was observed in non-chemotactic (Δ*cheA*) and chemotactic [wild-type (WT)] *L. agilis* strains using the microscopic agar-drop assay. Details of the assay are described in Supplementary Fig. S2. (**a**) Representative microscopic images of the *L. agilis* Δ*cheA* or WT cells near the agar drop with pH adjusted to 3.0 at 0 or 5 min. (**b**) The relative number of cells represents the ratio of cells near the agar drop at 5 min to that at 0 min. Values are represented as the mean + standard error (SE) (n = 4). Significant difference is indicated using an asterisk (*P < 0.01; Student’s t-test). (**c**) Time-course chemotactic responses of *L. agilis* BKN88 (WT) to pH values of 3.0, 5.0, 7.0, and 12.0. The relative number of cells represents the ratio of cells near the agar drop at each time point to that at 0 min. Values are represented as the mean ± SE (n = 6). Significant difference between the pH 7.0 and other pH values is indicated using an asterisk (*P < 0.05; Dunnett’s test). (**d**) Susceptibility of *L. agilis* to acid. Colony-forming units (CFUs) of *L. agilis* BKN88*, L. johnsonii* NRIC 0220 T, *L. reuteri* PTL371, and *L. acidophilus* NCFM incubated in MRS with pH adjusted to 3.0 at 37 °C were counted once every 30 min. Values are represented as mean ± standard deviation (SD) (n = 3).

*L. agilis* may respond to acidic environments in the presence of stomach acids or organic acids produced by the gut microbes to survive in the gastrointestinal tract. To examine the chemotaxis of *L. agilis* to low pH, chemotactic or non-chemotactic *L. agilis *strains were subjected to a microscopic agar-drop assay. Figure 1a, b shows that the non-chemotactic mutant (Δ*cheA*) did not respond significantly to the agar with a pH adjusted to 3.0 with HCl. In contrast, the chemotactic *L. agilis* (WT) escaped from the acidic agar. pH taxis have also been observed in some pathogenic bacteria, such as *E. coli*, *S. enterica,* and *H. pylori*^11,15,34–39^. *E. coli* exhibits repellent responses to acidic and basic pH conditions, resulting in its migration to neutral environments ^39^. *H. pylori* escapes acidic environments via attractant responses to basic pH and repellent responses to acidic pH ^40^. To examine these bidirectional responses of *L. agilis* to pH, the chemotactic responses of *L. agilis* to the agar with pH values ranging from 3.0 to 12.0 were analyzed. Chemotactic *L. agilis* responded only to acidic agar, whose pH was adjusted to 3.0 or 5.0 (Fig. 1c). This repellent response was stronger at lower pH (Fig. 1c). Several gut microbes, including lactobacilli, exhibit tolerance to acid^41,42^. To investigate the acid tolerance of *L. agilis*, *L. agilis* BKN88 and three strains of non-motile lactobacilli isolated from animals and humans were incubated under acidic conditions (pH 3.0), and the number of viable cells was counted over time. The sensitivity of *L. agilis* to acid was higher than that of the gut-derived non-motile lactobacilli (Fig. 1d). Some acid tolerance systems have been identified in non-motile lactobacilli ^42^. In *L. agilis*, only the F_1_F_0_-ATPase system, which plays a vital role in pH homeostasis in bacterial cells ^43^, was found among other systems (Supplementary Fig. S3). However, other systems, such as the malolactic fermentation (MLF) pathway (*mleRPS* genes), glutamate decarboxylase (GAD) system (*gadBC* genes), arginine deiminase (ADI) system (*arcABC* genes), and urease system (*ureABC* genes) were not detected in *L. agilis*.

### Chemotactic responses of *L. agilis* to bile

**Figure 2:**
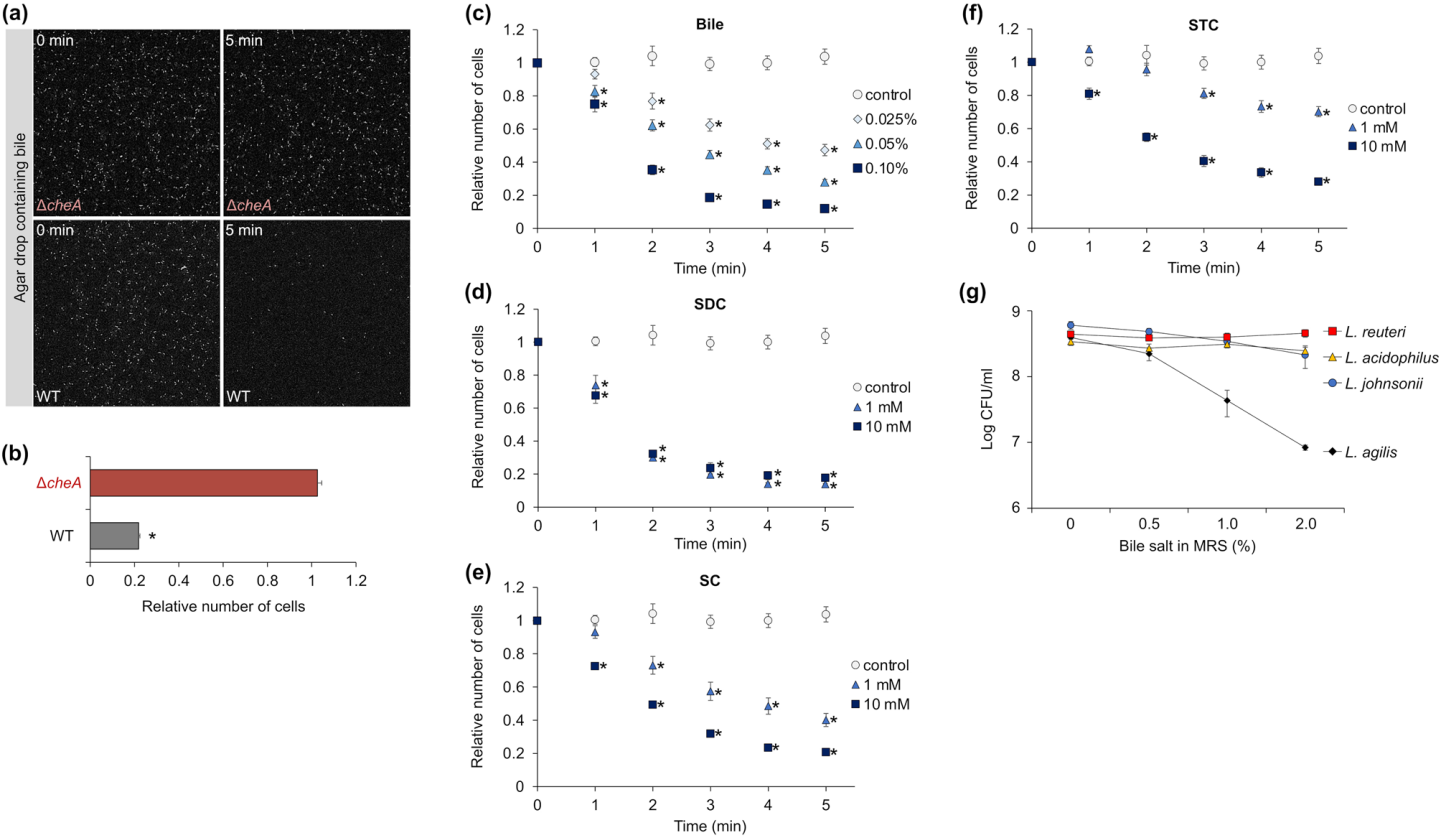
Chemotactic responses of *L. agilis* to bile. (**a**–**b**) Chemotaxis toward bile was observed in non-chemotactic (Δ*cheA*) and chemotactic (WT) *L. agilis* strains using the microscopic agar-drop assay. (**a**) Representative microscopic images of the *L. agilis* Δ*cheA* or WT cells near the agar drop containing 0.1% (w/v) bile salt at 0 or 5 min. (**b**) The relative number of cells represents the ratio of cells near the agar drop at 5 min to that at 0 min. Values are represented as the mean + SE (n = 4). Significant difference is indicated using an asterisk (*P < 0.01; Student’s t-test). (**c**–**f**) Time-course chemotactic responses of *L. agilis* BKN88 (WT) to various concentrations of bile salt (**c**) and its constituents [sodium deoxycholate (SDC), sodium cholate (SC), and sodium taurocholate (STC)]. The relative number of cells represents the ratio of cells near the agar drop at each time point to that at 0 min. An agar drop without test chemicals was used as a control. Values are represented as the mean ± SE (n = 6). Significant difference between the values of the control group and groups involving various concentrations of test chemicals is indicated using an asterisk (*P < 0.05; Dunnett’s test). (**g**) Susceptibility of *L. agilis* BKN88 to bile salt. CFUs of *L. agilis* BKN88, *L. johnsonii* NRIC 0220 T, *L. reuteri* PTL371, and *L. acidophilus* NCFM cultured on an MRS plate containing 0–2.0% (w/v) bile salts were counted. Values are represented as the mean ± SD (n = 3).

Bile exerts antimicrobial effects and is a major stressor for gut microbes. Several pathogens, including *H. pylori*, *Campylobacter jejuni*, and *Vibrio cholerae*, exhibit chemotaxis toward bile and its components^8,44–46^. Bile and some conjugated bile acids serve as repellents for *H. pylori*^8^, whereas bile and taurocholic acids serve as attractants for *V. cholerae* ^46^. To investigate bile chemotaxis in *L. agilis*, chemotactic and non-chemotactic *L. agilis* strains were subjected to a microscopic agar-drop assay. In contrast, to the non-chemotactic mutant, chemotactic *L. agilis* moved away from the bile salts (Fig. 2a, b) and exhibited dose-dependent repellent responses to bile salts (Fig. 2c). To identify the constituents of bile that elicit a repellent response, chemotaxis of *L. agilis* to major constituents of bile, such as sodium deoxycholate (SDC), sodium cholate (SC), and sodium taurocholate (STC), was examined. Because *L. agilis* exhibits repellent responses to low pH, the pH of the bile constituents tested in the microscopic agar-drop assay was adjusted to 7.0. Figure 2d–f shows that all tested bile constituents served as repellents. Among the bile constituents, the strongest and weakest repellent responses were elicited by SDC and STC, respectively. The repellent response to SC was between SDC and STC (Fig. 2d–f). Because these bile constituents are sodium salts, chemotaxis to sodium chloride was investigated. *L. agilis *did not exhibit chemotaxis toward NaCl (data not shown). The bile tolerance of *L. agilis* BKN88 and three non-motile lactobacillus strains was also examined. Although bile did not significantly inhibit the growth of non-motile lactobacilli, bile salt concentration-dependently decreased the viability of *L. agilis* BKN88 (Fig. 2g). The growth of *L. agilis* BKN88 was inhibited in the presence of high concentrations of bile salts [1.0–2.0% (w/v)]. The mechanisms involved in bile tolerance in gut-derived non-motile lactobacilli are mainly mediated by bile efflux, bile salt hydrolysis, and changes in cell membrane/wall architecture and composition^29,47^. Next, genes involved in bile tolerance in *L. agilis* BKN88 were examined. As shown in Supplementary Table S1, several putative genes encoding bile efflux pumps and bile salt hydrolases were identified in the genome of *L. agilis* BKN88.

### Chemotactic responses of *L. agilis* to organic acids produced by gut microbes

**Figure 3:**
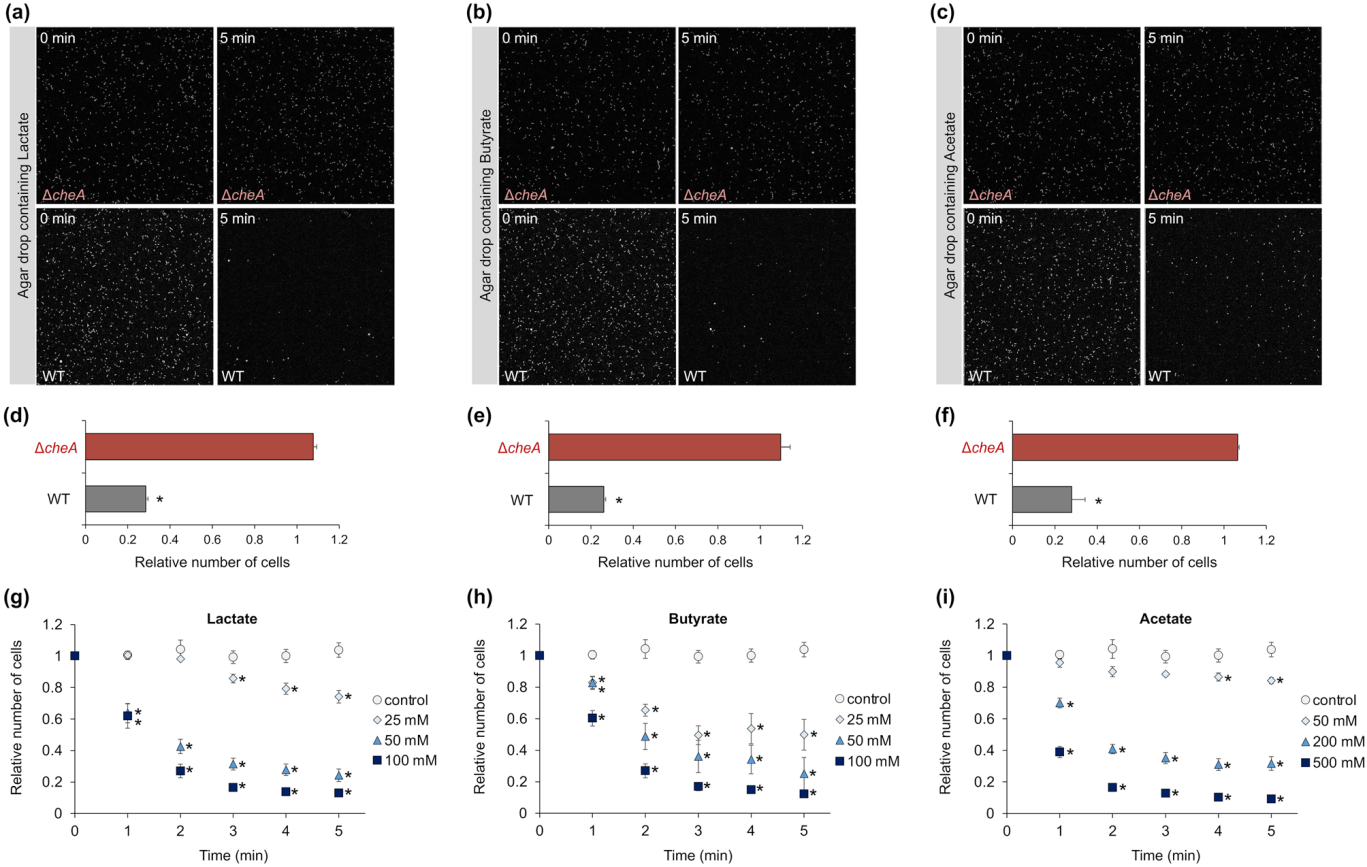
Chemotactic responses of *L. agilis* to organic acids produced by gut microbes. (**a**–**f**) Chemotaxis toward 100 mM lactate (**a**, **d**), 100 mM butyrate (**b**, **e**), and 500 mM acetate (**c**, **f**) were observed in non-chemotactic (Δ*cheA*) and chemotactic (WT) *L. agilis* strains using the microscopic agar-drop assay. (**a**–**c**) Representative microscopic images of the *L. agilis* Δ*cheA* or WT cells near the agar drop containing organic acid salts at 0 or 5 min. (**d**–**f**) The relative number of cells represents the ratio of cells near the agar drop at 5 min to that at 0 min. Values are represented as the mean + SE (n = 4). Significant difference is indicated using an asterisk (*P < 0.01; Student’s t-test). (**g**–**i**) Time-course chemotactic responses of *L. agilis* BKN88 (WT) to various concentrations of organic acid salts. The relative number of cells represents the ratio of cells near the agar drop at each time point to that at 0 min. An agar drop without test chemicals was used as a control. Values are represented as the mean ± SE (n = 6). Significant difference between the values of the control group and groups involving various concentrations of organic acid salts is indicated using an asterisk (*P < 0.05; Dunnett’s test).

Some organic acids, such as lactic acid, butyric acid, and acetic acid, are the main metabolites produced by gut microbes that compete with *L. agilis*. Therefore, in this study, we examined the chemotaxis of *L. agilis* in response to these organic acids. As *L. agilis* exhibits repellent responses to low pH, organic acid salts with a pH value adjusted to 7.0 were used to perform the microscopic agar-drop assay. The non-chemotactic mutant did not exhibit significant responses to organic acid salts, whereas the chemotactic strain showed repellent responses (Fig. 3a–f). Furthermore, the chemotactic response of *L. agilis* to various concentrations of organic acid salts was examined. The repellent responses to the organic acid salts were dose-dependent (Fig. 3g–i). A repellent response to acetate was only observed at high concentrations (> 50 mM) (Fig. 3i). It has been reported that most chemicals, such as various sugars and inorganic compounds, at high concentrations (> 1 M) serve as repellents for *E. coli*, which may be due to high osmolarity^48^. In contrast, *L. agilis* did not exhibit chemotactic responses to 500 mM d-galactose, 500 mM l-fucose, or 100 mM NaCl (data not shown) but was repelled by 25 mM lactate, 25 mM butyrate, and 50 mM acetate. Therefore, the repellent response of *L. agilis* to organic acids may not be attributable to high osmolarity.

### Chemotaxis-related genes and the features in* L. agilis* BKN88

**Figure 4:**
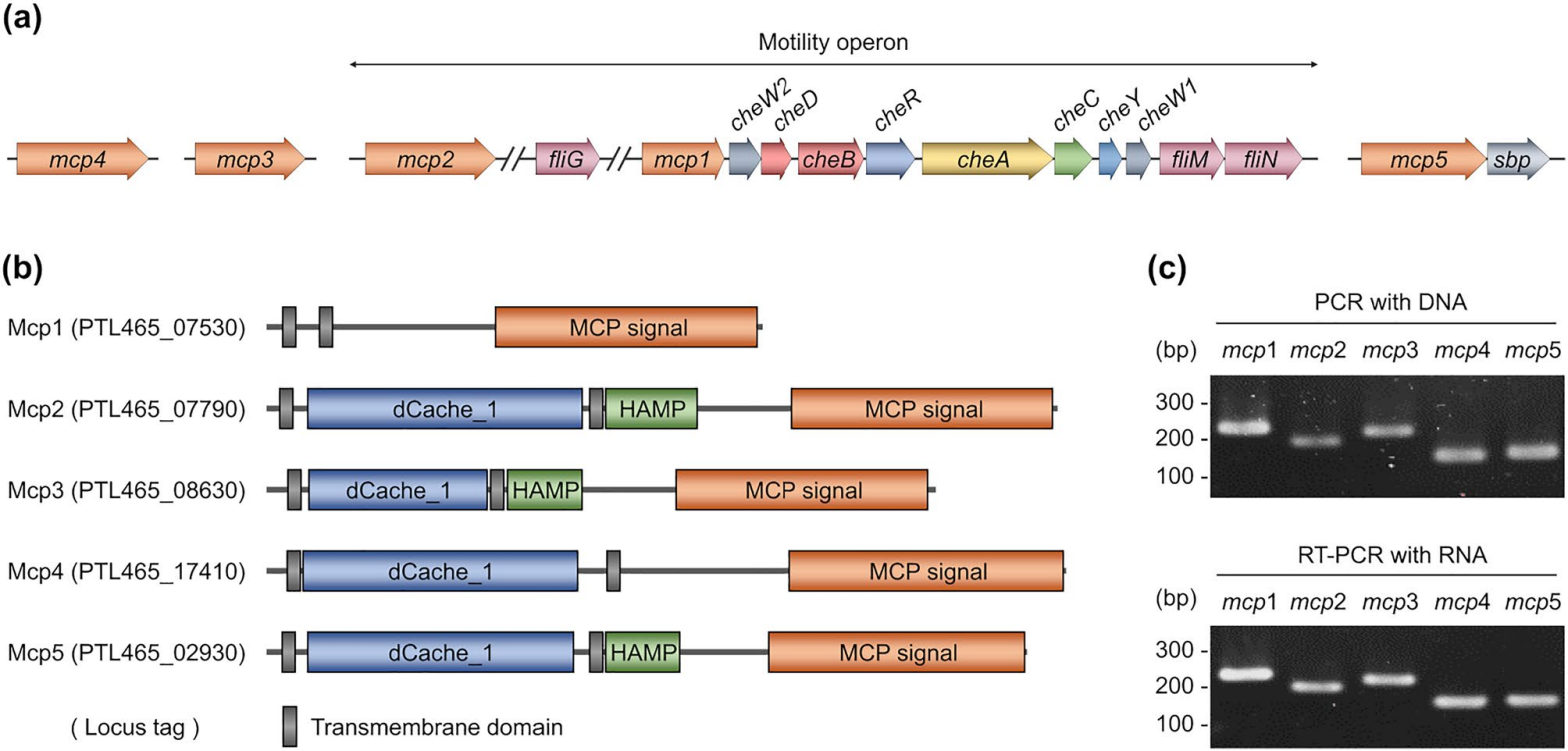
Chemoreceptors of *L. agilis* BKN88. (**a**) A genetic map of chemotaxis-related genes in the *L. agilis* BKN88 genome. (**b**) Domain architectures of the five chemoreceptors were predicted using InterPro^81^ and CDvist^82^. Transmembrane domains are shown as gray rectangles. MCP signal, cytoplasmic signaling domain; dCache_1 (a dual calcium channel and chemotaxis receptor), one of the ligand-binding domains; HAMP (histidine kinases, adenylyl cyclases, methyl binding proteins, and phosphatases), linker domain. (**c**) Expression of the MCP-encoding genes was evaluated using RT-PCR. PCR was performed with chromosomal DNA isolated from bacterial cells (upper). RT-PCR was performed with total RNA isolated from bacterial cells (lower).

As illustrated in Fig. 4a, the draft genome sequence analysis of *L. agilis* BKN88 revealed the presence of only one set of chemotaxis-related genes, except for two *cheW* genes, in a single gene cluster (motility operon) and five putative MCP-encoding genes (*mcp1–mcp5*). *mcp1* and *mcp2* are encoded in the motility operon, whereas *mcp3*, *mcp4*, and *mcp5* are encoded elsewhere in the genome. Chemoreceptors sense ligands directly or indirectly via interactions with periplasmic ligand-binding proteins (LBPs)^49^. Genes encoding LBPs are often located adjacent to chemoreceptor-encoding genes. In the genome of *L. agilis* BKN88, the LBP-encoding gene, which encodes the sugar-binding protein, was located in the vicinity of *mcp5* but not in the vicinity of *mcp1*, *mcp2*, *mcp3*, and *mcp4* (Figs. 4a and S4). Domain architecture analysis of the five chemoreceptors revealed that Mcp2, Mcp3, Mcp4, and Mcp5 have the following typical MCP domains: two transmembrane regions, a variable periplasmic ligand-binding domain (LBD), and a conserved cytoplasmic signaling domain. The LBDs of these four chemoreceptors contain a dual calcium channel and chemotaxis receptor (dCache_1) domain, which is the predominant extracellular sensory domain in bacteria^50^. In contrast, Mcp1 has two transmembrane regions and a cytoplasmic signaling domain but no identifiable LBD (Fig. 4b). The primary structures of the sensory domains (LBDs) are highly variable because they have evolved to recognize specific ligands. To gain insight into the correlation between chemoreceptors and their ligands, the amino acid sequences of the LBDs of Mcp2 (255 amino acids; residues 34–288), Mcp3 (154 amino acids; residues 39–192), Mcp4 (249 amino acids; residues 40–288), and Mcp5 (246 amino acids; residues 33–278) were analyzed using BLASTP with default settings and a cutoff of 45% identity and 90% query coverage. Although LBDs exhibiting high similarity (> 45% identity) to the LBDs of Mcp2 or Mcp3 were not detected, the LBD of Mcp4 was highly similar to that of the chemoreceptor from *Ligilactobacillus ruminis* (65% identity), which is the most prevalent *Lactobacillus* species in the human gut^51^. The LBD of Mcp5 shared sequence similarity with that of chemoreceptors from several butyrate-producing bacteria found in the human gut [belonging to the genera *Eubacterium*, *Lachnospira*, and *Roseburia* (approximately 45–50% identity)], as well as with that of *L. ruminis* (65% identity). The genes encoding sugar-binding proteins were also located near the genes encoding these chemoreceptors in the butyrate-producing bacteria and *L. ruminis*, similar to *mcp5* in *L. agilis* (Supplementary Fig. S4). The similarity between the LBDs of the four *L. agilis* chemoreceptors, except Mcp1, was less than 30%. Reverse transcription PCR (RT-PCR) analysis, performed to validate the transcription of chemoreceptor-encoding genes in *L. agilis* BKN88, revealed that all chemoreceptor genes were expressed during the exponential phase (Fig. 4c).

### Chemotactic responses of *L. agilis* mutants lacking one or all of the five MCP genes

**Figure 5:**
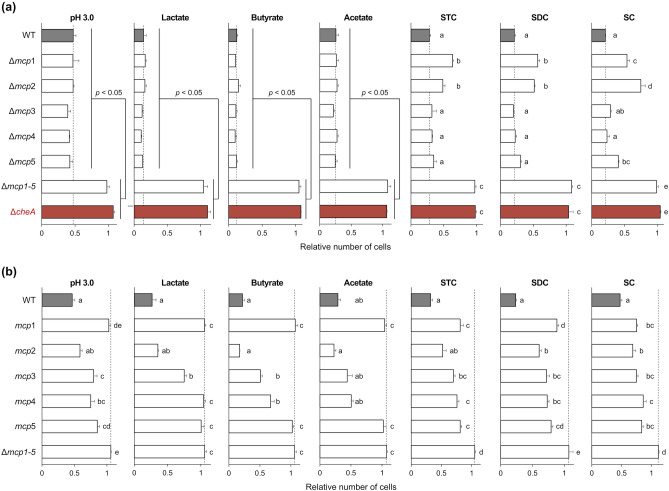
Chemotactic responses of the putative methyl-accepting chemotaxis protein (MCP) deletion mutants. (**a**–**b**) Chemotaxis toward pH 3.0, 100 mM lactate, 100 mM butyrate, 500 mM acetate, 20 mM sodium taurocholate (STC), 10 mM sodium deoxycholate (SDC), and 10 mM sodium cholate (SC) was observed in *L. agilis* strains lacking one or all of the MCP-encoding genes (**a**) and *L. agilis* strains expressing individual MCP-encoding genes (**b**). The relative number of cells represents the ratio of cells near the agar drop at 5 min to that at 0 min. Values are represented as the mean + SE (n = 6). Different superscripts indicate significant differences (*P* < 0.05; Tukey’s multiple comparison test). The dotted lines represent the values of *L. agilis* WT (**a**) or *L. agilis* Δ*mcp1-5* (**b**).

To identify the chemoreceptors responsible for sensing chemoeffectors in *L. agilis*, *L. agilis* mutants lacking one or all of the five putative MCP-encoding genes were constructed. PCR analysis using primers specific to the sequences flanking each MCP region revealed the deletion of MCP-encoding genes in *L. agilis* mutants (Supplementary Fig. S5a). The motility of MCP deletion mutants was also assessed in MRS soft-agar culture. In contrast, to the non-motile *L. agilis* BKN134 strain, all the MCP deletion mutants exhibited motility (Supplementary Fig. S5b). Next, the chemotactic responses of the MCP deletion mutants were determined using a microscopic agar drop assay. The *L. agilis* mutant deficient in all the five MCP-encoding genes (Δ*mcp1-5*) did not exhibit chemotactic responses to all tested chemoeffectors, while the *L. agilis* mutants lacking one of the five MCP-encoding genes showed repellent responses to chemoeffectors at the same levels as the WT *L. agilis*, except for the constituents of bile (Fig. 5a). These results indicate that multiple MCPs (Mcp1–Mcp5) may be involved in the chemotactic responses to low pH and organic acid salts in a complementary manner. Chemotactic responses to STC, SDC, and SC were attenuated in the Δ*mcp1* and Δ*mcp2* mutants. Meanwhile, chemotactic responses to SC were attenuated in the Δ*mcp5* mutants (Fig. 5a). These results suggest that Mcp1 and Mcp2 mediate chemotactic responses to STC, SDC, and SC and that Mcp5 is involved in chemotactic responses to SC.

### Chemotactic responses of* L. agilis* mutants expressing single MCP genes

**Figure 6:**
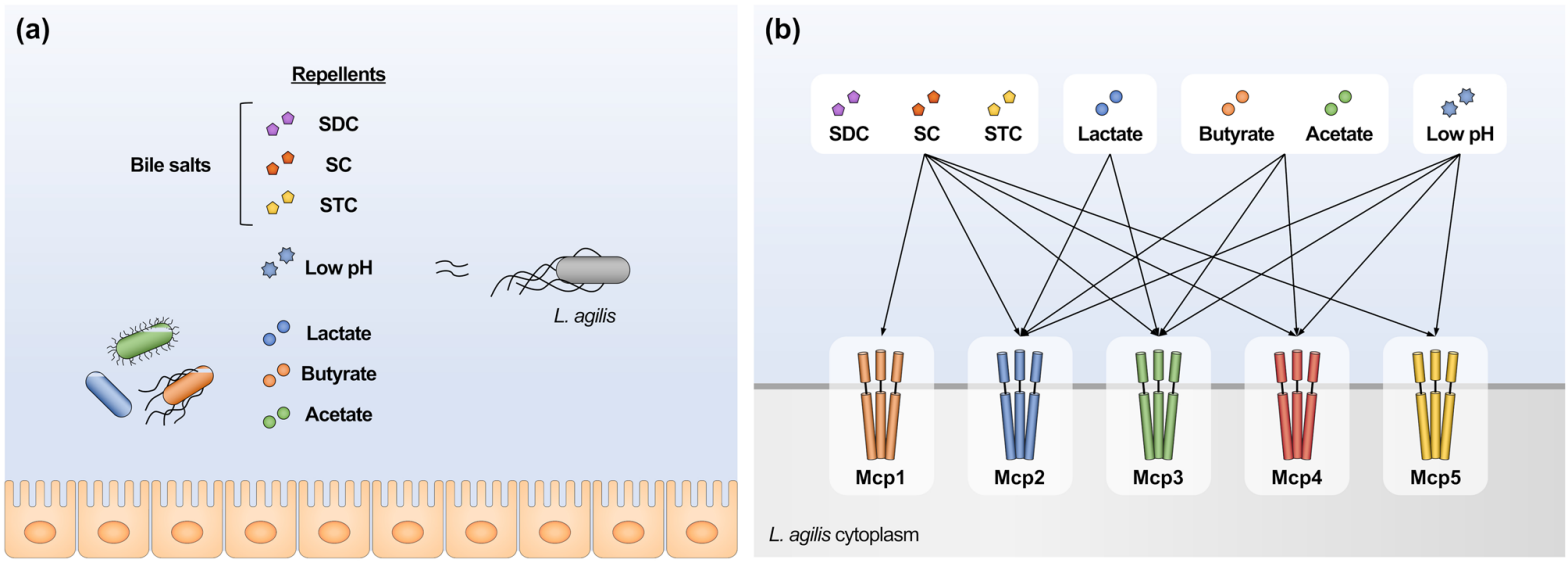
Schematic presentation of gut-derived chemoeffectors (**a**) and their corresponding chemoreceptors (**b**) in *L. agilis* identified in this study.

Next, *L. agilis* mutants expressing a single chemoreceptor were constructed. The chemotaxis of these mutants was examined to identify the chemoreceptors that sense chemoeffectors. *L. agilis* mutants expressing each of the five MCP-encoding genes were first constructed by introducing a plasmid carrying one of the MCP-encoding genes into the Δ*mcp1-5* mutant, which lacks all five MCP-encoding genes. However, the constructed mutants exhibit decreased motility, which hinders the observation of their chemotaxis. Thus, *L. agilis* mutants expressing a single MCP-encoding gene with four other MCPs deleted were constructed (Supplementary Fig. S6). As shown in Fig. 5b, the microscopic agar-drop assay with these mutants revealed that the mutant with Mcp1 as the sole chemoreceptor was defective in chemotaxis to low pH, and organic acid salts could swim away from the three tested bile constituents. This was consistent with the results of the chemotaxis assay performed using the Δ*mcp1* mutant (Fig. 5a). *L. agilis* mutants expressing only Mcp2 or Mcp3 exhibited repellent responses to all tested chemoeffectors. Among the mutants, the Mcp2-expressing strain exhibited the strongest repellent response to all chemoeffectors. This suggests that Mcp2 is a major chemoreceptor of gut-derived substances in *L. agilis*. Mutant strains expressing Mcp4 or Mcp5 exhibited chemotaxis to all chemoeffectors except for lactate or organic acid salts, respectively. These data suggest that each of the five MCPs responds to some or all of the chemoeffectors and that these MCPs are redundantly involved in chemotaxis to each chemoeffectors (Fig. 6).

## Discussion

We previously reported that *L. agilis* BKN88 colonized the gut in murine models via motility behavior^31^. This suggests that chemotaxis is also involved in gut colonization by *L. agilis*. The role of chemotaxis in host colonization has been demonstrated in some pathogenic bacteria^24^. However, few studies have examined the role of chemotaxis in the gut colonization of beneficial gut microbes, such as lactobacilli, owing to a lack of information on their chemoeffectors and chemoreceptors. In this study, we performed a chemotaxis assay using chemotactic and non-chemotactic *L. agilis* strains and a series of chemoreceptor mutants to identify chemoeffectors and their corresponding chemoreceptors in *L. agilis* BKN88.

Gut microbes sense and respond to gastrointestinal stress conditions, such as acidic and bile stress, to survive under harsh conditions in the gastrointestinal tract. Some pathogens, including *E. coli*, *S. enterica, H. pylori*, and *C. jejuni*, escape low pH and bile via motility and chemotaxis ^8,11,15,34–39,44,45^. Chemotaxis has been reported to be involved in host colonization and cell invasion in these bacteria ^24,52–59^. This study demonstrated that *L. agilis* BKN88 swims away from low pH conditions and bile. This suggests that *L. agilis*, a commensal microbe, survives and persists in the gastrointestinal tract via repellent responses to acids and bile. *L. agilis* is more sensitive to acid and bile than gut-derived non-motile lactobacilli. Therefore, the repellent response to these stress conditions may be an important factor for the survival of *L. agilis* in the gut. These findings suggest that stress tolerance is not essential for *L. agilis*, which can escape acids and bile via motility and chemotaxis. Several gut microbes, including lactobacilli, exhibit acid and bile tolerance^41,47^, whereas *L. agilis* likely utilizes motility and chemotaxis instead of stress tolerance/resistance. This also hypothesizes that lactobacilli might have chosen one of the following two evolutionary strategies: establishing a sedentary lifestyle that does not require motility or a nomadic lifestyle based on motility and chemotaxis.

*L. agilis *BKN88 exhibited repellent responses to organic acids, lactate, acetate, and butyrate. Organic acids are the main metabolites produced by gut microbes and exert toxic effects on bacterial cells at high concentrations. In addition, high acid concentrations can lead to decreased pH in the intestine, resulting in bacterial growth inhibition^60,61^. Hence, the chemotactic response to organic acids is important for escape from toxic acids and a low pH environment and may confer survival advantages in the intestine. Gut microbes that produce organic acids compete with *L. agilis*. Therefore, another possible benefit of chemotactic responses to organic acids may be the ability to escape competitive gut microbiota, resulting in their migration to a specific niche in the gastrointestinal tract. Previously, we demonstrated that the effect of the presence or absence of motility on gut colonization by *L. agilis* in specific pathogen-free mice was higher than that in germ-free mice^31^. This finding suggests that motility is beneficial when *L. agilis* is surrounded by other gut microbes. In this case, the benefits of motility may be attributed to the repellent responses to organic acids and their producers. Thus, the repellent response to organic acids may play an important role in the survival and competition of *L. agilis* in the gut.

*L. agilis *BKN88 contains five putative chemoreceptor genes (*mcp1–mcp5*). RT-PCR analysis confirmed the expression of these genes in *L. agilis* BKN88. A chemotaxis assay with chemoreceptor deletion mutants revealed that the single MCP deletion mutants exhibited repellent responses to the chemoeffectors. In contrast, mutants deficient in all five MCP-encoding genes did not exhibit repellent responses to all tested chemoeffectors. These results indicate that the chemoreceptors of *L. agilis* are functionally redundant and may be involved in chemotactic responses to chemoeffectors in a complementary manner. This is consistent with the results of a chemotaxis assay using mutants expressing a single chemoreceptor, in which chemotaxis to each effector was observed in some or all mutants. Functional redundancy of chemoreceptors has also been described in many bacteria and may be a common survival strategy for motile bacteria in their environment^6^. However, the need for multiple chemoreceptors to detect the same ligands in bacteria remains unclear. One possible reason for this redundancy is that the chemotaxis of bacteria is not impaired, even if there is a loss or mutation of any chemoreceptor. The chemotaxis of *L. agilis* to chemoeffectors was not completely impaired in *L. agilis* mutants lacking one of the five chemoreceptors. In particular, the chemotaxis of the mutants in response to low pH and organic acids was comparable to that of the WT. Thus, the functional redundancy of *L. agilis* chemoreceptors for gut-derived substances may contribute to the robust chemotactic ability of *L. agilis* in the environment.

The findings of this study also indicate that each of the five chemoreceptors in *L. agilis* can sense multiple chemoeffectors. Mcp2, Mcp3, and Mcp4, which are involved in the responses to low pH, some organic acids, and bile components, may sense a broad range of ligands. Domain architecture analysis of the chemoreceptors revealed that the LBDs (sensing domains) of *L. agilis* chemoreceptors, except for Mcp1, contained a dCache_1 domain. Several dCache-containing chemoreceptors can sense multiple ligands^62–68^. Our findings are consistent with those of previous studies.

Several chemoreceptors of the chemoeffectors tested in this study have been previously reported (Supplementary Table S2). To our knowledge, this is the first study to report chemoreceptors in the SC. Mcp2, Mcp3, and Mcp4 are the only identified dCache-containing chemoreceptors for butyrate and acetate. Therefore, the findings of this study may provide additional information on bacterial chemoreceptor-ligand interactions. However, the affinity of chemoreceptors for effectors and the ability of the receptors to directly sense the effectors are unknown. Thus, further studies (e.g., isothermal titration calorimetry analysis) are required. Some motile bacteria monitor their intracellular energy levels via chemoreceptors and migrate to favorable niches for energy generation^69,70^. This bacterial behavior is known as energy taxis, and the effectors of energy taxis include electron acceptors, light, redox chemicals, and metabolizable substrates. For example, *E. coli* senses the proton motive force and the redox state of the electron transport system via two chemoreceptors, Tsr and Aer^71,72^. In *H. pylori*, low pH, which directly influences the proton motive force, and changes in the electron transport chain are sensed by TlpB and TlpD, respectively^15,73^. Although the chemotactic behavior of *L. agilis* is not completely understood, further studies may reveal that it includes energy taxis.

In conclusion, this study demonstrated that *L. agilis*, which is highly sensitive to gut-associated stress conditions, escapes the stress and metabolites of competitive gut microbiota via chemotaxis. This suggests that *L. agilis* can utilize chemotaxis to facilitate its survival and persistence in the gastrointestinal tract. Multiple *L. agilis* chemoreceptors mediate the chemotaxis of each gut-derived substance in a complementary manner. This functional redundancy of chemoreceptors may confer a robust chemotactic ability to *L. agilis* in the environment. The findings of this study provide further insights into the interactions between chemoreceptors and ligands of motile lactobacilli and the unique ecological features and evolutionary strategies of motile lactobacilli, which may be distinct from those of non-motile lactobacilli.

## Methods

### Bacterial strains and growth conditions

The bacterial strains and plasmids used in this study are listed in Table 1. *L. agilis *BKN88 ^74^ and its derivative strains were anaerobically propagated in MRS broth or agar (Difco, BD, USA) with or without 5 μg mL^−1^ of erythromycin at 37 °C. The motility of *L. agilis* strains was determined by observing strains cultured in a semi-solid MRS medium with 0.3% agar. Bacterial motility was observed using a BZ-X710 microscope (KEYENCE, Osaka, Japan). *E. coli* EC101 and derivative strains were aerobically grown in Brain Heart Infusion (Difco, BD, USA) broth or agar with or without 200 μg mL^−1^ of erythromycin and 40 μg mL^−1^ of kanamycin at 37 °C.

**Table 1:** Bacterial strains and a plasmid used in this study.

Strain or plasmid	Description and Origin	References
*E. coli*		
EC101	Cloning host for pG^+^host5, RepA^+^	^78^
*L. agilis*		
BKN88 (WT)	Motile subculture of JCM 1048, Chicken isolate	^74^
PTL728 (Δ*cheA*)	BKN88 Δ*cheA*	This study
PTL452 (Δ*mcp1*)	BKN88 Δ*mcp1*	This study
PTL650 (Δ*mcp2*)	BKN88 Δ*mcp2*	This study
PTL651 (Δ*mcp3*)	BKN88 Δ*mcp3*	This study
PTL652 (Δ*mcp4*)	BKN88 Δ*mcp4*	This study
PTL679 (Δ*mcp5*)	BKN88 Δ*mcp5*	This study
PTL766 (Δ*mcp1-5*)	BKN88 Deletion of all five putative *mcp* genes	This study
PTL854 (*mcp1*)	BKN88 Δ*mcp2* Δ*mcp3* Δ*mcp4* Δ*mcp5*	This study
PTL804 (*mcp2*)	BKN88 Δ*mcp1* Δ*mcp3* Δ*mcp4* Δ*mcp5*	This study
PTL802 (*mcp3*)	BKN88 Δ*mcp1* Δ*mcp2* Δ*mcp4* Δ*mcp5*	This study
PTL798 (*mcp4*)	BKN88 Δ*mcp1* Δ*mcp2* Δ*mcp3* Δ*mcp5*	This study
PTL739 (*mcp5*)	BKN88 Δ*mcp1* Δ*mcp2* Δ*mcp3* Δ*mcp4*	This study
*L. acidophilus* NCFM	Isolated from the human intestine	^79^
*L. reuteri* PTL371	Isolated from murine feces	This study
*L. johnsonii* NRIC 0220^ T^	Isolated from human	NRIC
Plasmid		
pG^+^host5	Replication-thermo-sensitive plasmid, Em^r^	^80^

### Microscopic agar-drop assay

The microscopic agar-drop assay was performed according to the protocols described by Islarm et al.^33^ with minor modifications (Supplementary Fig. S2). A flow chamber containing an agar drop was prepared as follows: Test reagent in distilled water containing 1.5% (w/v) agar was dropped onto the center of a long cover glass (NEO Micro Cover Glass, 24 × 60 mm, No. 1:0.12–0.17 mm in thickness, Matsunami, Osaka, Japan), followed by the attachment of a small cover glass (Micro Cover Glass, 18 × 18 mm, No. 1:0.12–0.17 mm in thickness, Matsunami, Osaka Japan) to the long cover glass using double-sided tape. The agar drop in the flow chamber was washed with chemotaxis buffer (10 mM potassium phosphate, 1 mM glucose, and 0.1 mM EDTA in pure water, pH 7.0). Bacterial cells in the exponential phase were harvested via centrifugation at 3900 rpm at 25 °C for 5 min and suspended in an MRS medium. The bacterial suspension was diluted 1:15 with a chemotaxis buffer and infused into the flow chamber. Motile cells near the agar drop were immediately observed, and images were captured using a time-lapse microscope (10 × objective, BZ-X710, Keyence, Osaka, Japan) once every 1 min. As shown in Supplementary Fig. S2, the number of cells in the red dotted area (500 µm × 500 µm area) was counted using ImageJ software (National Institutes of Health, Bethesda, MD, USA)^75^. The relative number of cells represents the ratio of the number of cells in the area at each time point to the number at 0 min. An agar drop without the test chemicals was used as a control.

### Test chemicals

The following chemicals were used in the chemotaxis assay: bile salts (Sigma‐Aldrich, USA), SDC (FUJIFILM Wako Pure Chemical Corporation, Osaka, Japan), SC (FUJIFILM Wako Pure Chemical Corporation, Osaka, Japan), STC (FUJIFILM Wako Pure Chemical Corporation, Osaka, Japan), mucin from the porcine stomach (Type II, Sigma-Aldrich, USA), lactic acid (FUJIFILM Wako Pure Chemical Corporation, Osaka, Japan), butyric acid (Tokyo Chemical Industry Co., Ltd, Tokyo, Japan), and acetic acid (FUJIFILM Wako Pure Chemical Corporation, Osaka, Japan). The pH of the test reagents used in the chemotaxis assays (excluding the pH taxis assay) was adjusted to 7.0 with HCl or NaOH solution. In the pH taxis assay, the pH of the agar drop was adjusted to 3.0, 5.0, 7.0, or 12.0, using HCl or NaOH.

### Expression analysis of MCP-encoding genes

Transcription of the five putative MCP-encoding genes was determined by RT-PCR using the specific primers listed in Supplementary Table S2. Total RNA was isolated from *L. agilis* BKN88 in the exponential phase using NucleoSpin RNA (Macherey–Nagel, Germany), following the manufacturer’s instructions. To digest the contaminated genomic DNA, RNA samples were treated with deoxyribonuclease (RT grade, Nippon Gene, Tokyo, Japan). RT-PCR was performed using a PrimeScript One Step RT-PCR Kit (Takara, Japan). DNA contamination in the RNA samples was tested by PCR using the same primers as those used for RT-PCR and Ex. Taq DNA polymerase (Takara, Japan).

### Construction of MCP-encoding or CheA-encoding gene deletion mutants

Putative MCP-encoding or CheA-encoding genes in *L. agilis* BKN88 were deleted via double-crossover using the thermosensitive plasmid pG^+^host5. The DNA fragments of the upstream and downstream regions of the target gene were amplified by PCR using the primers listed in Supplementary Table S3. The amplicons were fused by overlap PCR. The resulting PCR products were digested with appropriate restriction endonucleases, ligated into a similarly digested pG^+^host5 vector, and transformed into *E. coli* EC101 as the cloning host. Transformants harboring plasmids with the desired insert were selected using colony PCR and Sanger sequencing with the M13 primers DOKJ78 (5′-GTAAAACGACGGCCAGT-3′) and DOKJ79 (5′-CAGGAAACAGCTATGAC-3′). The constructed plasmids were introduced into *L. agilis* BKN88 via electroporation as described previously^31^. Erythromycin-resistant transformants were selected and cultured in MRS containing erythromycin at 28 °C. To integrate the plasmids into the genome, the transformants were then sub-cultured in MRS containing erythromycin at 42 °C, plated on MRS agar plates containing erythromycin, and incubated at 42 °C until colonies were visible. PCR confirmed the integration. The integrants were cultured in MRS without erythromycin at 37 °C, followed by seven to nine transfers in MRS without erythromycin. After plating the bacterial cultures on MRS agar plates, single colonies were replicated on MRS agar plates and MRS agar plates containing erythromycin. Deletion of target genes in erythromycin-sensitive transformants was validated by PCR using the primers listed in Supplementary Table S3.

### Analysis of swimming behavior

The swimming behavior of the WT and non-chemotactic (Δ*cheA*) *L. agilis* strains at the exponential phase was observed. Individual motile cells (n = 10) were tracked for 10 s (15 frames per second) under a BZ-X710 microscope (10 × objective; Keyence, Osaka, Japan) using video editing analysis software VW-H2MA (Keyence, Osaka, Japan). All coordinates (x,y) of the starting point of each track were set to (0,0).

### Bile tolerance

The bile tolerances of *L. agilis* BKN88 and the three non-motile *Lactobacillus* species listed in Table 1 were examined as previously described, with minor modifications^76^. Bacterial cells from the exponential phase were serially diluted, plated onto MRS agar plates containing 0%, 0.25%, 0.5%, 1.0%, and 2.0% (w/v) oxgall (Difco, USA), and incubated at 37 °C anaerobically for enumeration. Oxgall concentrations were used to simulate intestinal conditions.

### Acid tolerance

The acid tolerance of *L. agilis* BKN88 and the three non-motile *Lactobacillus* species listed in Table 1 was examined following the method described by Liong and Shah^77^. Bacterial cells from the exponential phase were suspended in MRS broth with pH adjusted to 3.0, with HCl, and incubated at 37 °C for 120 min. The bacterial cells were collected once every 30 min, plated onto MRS agar plates, and incubated at 37 °C anaerobically for enumeration.

### Supplementary Information

Supplementary Information.

## Data Availability

All data described in the manuscript are presented in the main text, figures, and Supporting Information.

## References

[1] Adler J (2008). Chemotaxis in bacteria motile *Escherichia*
*coli* migrate in bands that are influenced by oxygen and organic nutrients. Adv. Sci..

[2] Wadhams GH, Armitage JP (2004). Making sense of it all: Bacterial chemotaxis. Nat. Rev. Mol. Cell Biol..

[3] Porter SL, Wadhams GH, Armitage JP (2011). Signal processing in complex chemotaxis pathways. Nat. Rev. Microbiol..

[4] Parkinson JS, Hazelbauer GL, Falke JJ (2015). Signaling and sensory adaptation in *Escherichia*
*coli* chemoreceptors: 2015 update. Trends Microbiol..

[5] Sourjik V, Wingreen NS (2012). Responding to chemical gradients: Bacterial chemotaxis. Curr. Opin. Cell Biol..

[6] Ortega Á, Zhulin IB, Krell T (2017). Sensory repertoire of bacterial chemoreceptors. Microbiol. Mol. Biol. Rev..

[7] Hazelbauer GL, Falke JJ, Parkinson JS (2008). Bacterial chemoreceptors: High-performance signaling in networked arrays. Trends Biochem. Sci..

[8] Worku ML, Karim QN, Spencer J, Sidebotham RL (2004). Chemotactic response of *Helicobacter*
*pylori* to human plasma and bile. J. Med. Microbiol..

[9] Mizote T, Yoshiyama H, Nakazawa T (1997). Urease-independent chemotactic responses of *Helicobacter*
*pylori* to urea, urease inhibitors, and sodium bicarbonate. Infect. Immun..

[10] Huang JY (2015). Chemodetection and destruction of host urea allows *Helicobacter*
*pylori* to locate the epithelium. Cell Host Microbe.

[11] Goers Sweeney E (2012). Structure and proposed mechanism for the pH-sensing *Helicobacter*
*pylori* chemoreceptor TlpB. Structure.

[12] Machuca MA (2017). *Helicobacter*
*pylori* chemoreceptor TlpC mediates chemotaxis to lactate. Sci. Rep..

[13] Cerda O, Rivas A, Toledo H (2003). *Helicobacter*
*pylori* strain ATCC700392 encodes a methyl-accepting chemotaxis receptor protein (MCP) for arginine and sodium bicarbonate. FEMS Microbiol. Lett..

[14] Yoshiyama H, Nakamura H, Kimoto M, Okita K, Nakazawa T (1999). Chemotaxis and motility of *Helicobacter*
*pylori* in a viscous environment. J. Gastroenterol..

[15] Croxen MA, Sisson G, Melano R, Hoffman PS (2006). The *Helicobacter*
*pylori* chemotaxis receptor tlpB (HP0103) is required for pH taxis and for colonization of the gastric mucosa. J. Bacteriol..

[16] Sanders L, Andermann TM, Ottemann KM (2013). A supplemented soft agar chemotaxis assay demonstrates the *Helicobacter*
*pylori* chemotactic response to zinc and nickel. Microbiology (United Kingdom).

[17] Collins KD (2016). The *Helicobacter*
*pylori* CZB cytoplasmic chemoreceptor TlpD forms an autonomous polar chemotaxis signaling complex that mediates a tactic response to oxidative stress. J. Bacteriol..

[18] Rader BA (2011). *Helicobacter*
*pylori* perceives the quorum-sensing molecule AI-2 as a chemorepellent via the chemoreceptor TlpB. Microbiology.

[19] Johnson KS, Ottemann KM (2018). Colonization, localization, and inflammation: the roles of *H*. *pylori* chemotaxis in vivo. Curr. Opin. Microbiol..

[20] Keilberg D, Ottemann KM (2016). How *Helicobacter*
*pylori* senses, targets and interacts with the gastric epithelium. Environ. Microbiol..

[21] Glekas GD (2012). The *Bacillus*
*subtilis* chemoreceptor McpC senses multiple ligands using two discrete mechanisms. J. Biol. Chem..

[22] Hanlon DW, Ordal GW (1994). Cloning and characterization of genes encoding methyl-accepting chemotaxis proteins in *Bacillus*
*subtilis*. J. Biol. Chem..

[23] Müller J, Schiel S, Ordal GW, Saxild HH (1997). Functional and genetic characterization of mcpC, which encodes a third methyl-accepting chemotaxis protein in *Bacillus*
*subtilis*. Microbiology.

[24] Matilla MA, Krell T (2018). The effect of bacterial chemotaxis on host infection and pathogenicity. FEMS Microbiol. Rev..

[25] Cousin FJ (2015). Detection and genomic characterization of motility in *Lactobacillus*
*curvatus*: Confirmation of motility in a species outside the *Lactobacillus*
*salivarius* clade. Appl. Environ. Microbiol..

[26] Suzuki S (2020). PCR-based screening, isolation, and partial characterization of motile lactobacilli from various animal feces. BMC Microbiol..

[27] Gaucher F (2019). Review: Adaptation of beneficial propionibacteria, lactobacilli, and bifidobacteria improves tolerance toward technological and digestive stresses. Front. Microbiol..

[28] Goh YJ, Klaenhammer TR (2014). Insights into glycogen metabolism in *Lactobacillus*
*acidophilus*: Impact on carbohydrate metabolism, stress tolerance and gut retention. Microb. Cell Fact..

[29] Ruiz L, Margolles A, Sánchez B (2013). Bile resistance mechanisms in *Lactobacillus* and *Bifidobacterium*. Front. Microbiol..

[30] Duar RM (2017). Lifestyles in transition: Evolution and natural history of the genus *Lactobacillus*. FEMS Microbiol. Rev..

[31] Kajikawa A, Suzuki S, Igimi S (2018). The impact of motility on the localization of *Lactobacillus*
*agilis* in the murine gastrointestinal tract. BMC Microbiol..

[32] Rao CV, Kirby JR, Arkin AP (2004). Design and diversity in bacterial chemotaxis: A comparative study in *Escherichia*
*coli* and *Bacillus*
*subtilis*. PLoS Biol..

[33] Islam MS, Takabe K, Kudo S, Nakamura S (2014). Analysis of the chemotactic behaviour of *Leptospira* using microscopic agar-drop assay. FEMS Microbiol. Lett..

[34] Larsen SH, Reader RW, Kort EN, Tso WW, Adler J (1974). Change in direction of flagellar rotation is the basis of the chemotactic response in *Escherichia*
*coli*. Nature.

[35] Krikos A, Conley MP, Boyd A, Berg HC, Simon MI (1985). Chimeric chemosensory transducers of *Escherichia*
*coli*. Proc. Natl. Acad. Sci. U. S. A..

[36] Kihara M, Macnab RM (1981). Cytoplasmic pH mediated pH taxis and weak-acid repellent taxis of bacteria. J. Bacteriol..

[37] Repaske DR, Adler J (1981). Change in intracellular pH of *Escherichia*
*coli* mediates the chemotactic response to certain attractants and repellents. J. Bacteriol..

[38] Umemura T, Matsumoto Y, Ohnishi K, Homma M, Kawagishi I (2002). Sensing of cytoplasmic pH by bacterial chemoreceptors involves the linker region that connects the membrane-spanning and the signal-modulating helices. J. Biol. Chem..

[39] Yang Y, Sourjik V (2012). Opposite responses by different chemoreceptors set a tunable preference point in *Escherichia*
*coli* pH taxis. Mol. Microbiol..

[40] Huang JY, Goers Sweeney E, Guillemin K, Amieva MR (2017). Multiple acid sensors control helicobacter pylori colonization of the stomach. PLoS Pathog..

[41] Van de Guchte M (2002). Stress responses in lactic acid bacteria. Antonie van Leeuwenhoek Int. J. Gen. Mol. Microbiol..

[42] Papadimitriou K (2016). Stress physiology of lactic acid bacteria. Microbiol. Mol. Biol. Rev..

[43] Kullen MJ, Klaenhammer TR (1999). Identification of the pH-inducible, proton-translocating F 1 F 0 -ATPase (atpBEFHAGDC) operon of Lactobacillus acidophilus by differential display: Gene structure, cloning and characterization. Mol. Microbiol..

[44] Hugdahl MB, Beery JT, Doyle MP (1988). Chemotactic behavior of Campylobacter jejuni. Infect. Immun..

[45] Li Z (2014). Methyl-accepting chemotaxis proteins 3 and 4 are responsible for Campylobacter jejuni chemotaxis and jejuna colonization in mice in response to sodium deoxycholate. J. Med. Microbiol..

[46] Nishiyama SI (2016). Identification of a *Vibrio*
*cholerae* chemoreceptor that senses taurine and amino acids as attractants. Sci. Rep..

[47] Begley M, Gahan CGM, Hill C (2005). The interaction between bacteria and bile. FEMS Microbiol. Rev..

[48] Li C, Boileau AJ, Kung C, Adler J (1988). Osmotaxis in *Escherichia*
*coli*. Proc. Natl. Acad. Sci. U. S. A..

[49] Matilla MA, Ortega Á, Krell T (2021). The role of solute binding proteins in signal transduction. Comput. Struct. Biotechnol. J..

[50] Upadhyay AA, Fleetwood AD, Adebali O, Finn RD (2016). Cache domains that are homologous to, but different from PAS domains comprise the largest superfamily of extracellular sensors in prokaryotes. PLoS Comput. Biol..

[51] Ghosh TS (2020). Metagenomic analysis reveals distinct patterns of gut lactobacillus prevalence, abundance, and geographical variation in health and disease. Gut Microbes.

[52] Szymanski CM, King M, Haardt M, Armstrong GD (1995). Campylobacter jejuni motility and invasion of Caco-2 cells. Infect. Immun..

[53] Golden NJ, Acheson DWK (2002). Identification of motility and autoagglutination *Campylobacter*
*jejuni* mutants by random transposon mutagenesis. Infect. Immun..

[54] O’Toole PW, Lane MC, Porwollik S (2000). Helicobacter pylori motility. Microbes Infect..

[55] Aihara E (2014). Motility and chemotaxis mediate the preferential colonization of gastric injury sites by *Helicobacter*
*pylori*. PLoS Pathog..

[56] Josenhans C, Suerbaum S (2002). The role of motility as a virulence factor in bacteria. Int. J. Med. Microbiol..

[57] Andino A, Hanning I (2015). *Salmonella*
*enterica*: Survival, colonization, and virulence differences among serovars. Sci. World J..

[58] Millet YA (2014). Insights into *Vibrio*
*cholerae* intestinal colonization from monitoring fluorescently labeled bacteria. PLoS Pathog..

[59] Butler SM, Camilli A (2004). Both chemotaxis and net motility greatly influence the infectivity of *Vibrio*
*cholerae*. Proc. Natl. Acad. Sci. U. S. A..

[60] Ricke SC (2003). Perspectives on the use of organic acids and short chain fatty acids as antimicrobials. Poult. Sci..

[61] Sun, Y. & O’Riordan, M. X. D. Chapter three - regulation of bacterial pathogenesis by intestinal short-chain fatty acids. In (eds. Sariaslani, S. & Gadd, G. M. B. T.-A. in A. M.) vol **85**, 93–118 (Academic Press, 2013). 10.1016/B978-0-12-407672-3.00003-4 PMC4029053 23942149

[62] Feng H (2022). Signal binding at both modules of its dCache domain enables the McpA chemoreceptor of *Bacillus*
*velezensis* to sense different ligands. Proc. Natl. Acad. Sci..

[63] Ud-din AIMS, Khan MF, Roujeinikova A (2020). Broad specificity of amino acid chemoreceptor CtaA of *pseudomonas*
*fluorescens* is afforded by plasticity of its amphipathic ligand-binding pocket. Mol. Plant-Microbe Interact..

[64] Webb BA, Compton KK, Ray WK, Helm RF, Scharf BE (2017). *Sinorhizobium*
*meliloti* chemotaxis to quaternary ammonium compounds is mediated by the chemoreceptor McpX. Mol. Microbiol..

[65] Fernández M, Morel B, Corral-Lugo A, Krell T (2016). Identification of a chemoreceptor that specifically mediates chemotaxis toward metabolizable purine derivatives. Mol. Microbiol..

[66] Gavira JA (2018). Structural basis for polyamine binding at the dCACHE domain of the McpU Chemoreceptor from *Pseudomonas*
*putida*. J. Mol. Biol..

[67] Corral Lugo A (2018). High-Affinity Chemotaxis to Histamine Mediated by the TlpQ chemoreceptor of the human pathogen *Pseudomonas*
*aeruginosa*. MBio.

[68] Johnson KS (2021). The dCache chemoreceptor TlpA of helicobacter pylori binds multiple attractant and antagonistic ligands via distinct sites. MBio.

[69] Alexandre G, Greer-Phillips S, Zhulin IB (2004). Ecological role of energy taxis in microorganisms. FEMS Microbiol. Rev..

[70] Josenhans C, Schweinitzer T (2010). Bacterial energy taxis: A global strategy?. Arch. Microbiol..

[71] Rebbapragada A (1997). The Aer protein and the serine chemoreceptor Tsr independently sense intracellular energy levels and transduce oxygen, redox, and energy signals for *Escherichia*
*coli* behavior. Proc. Natl. Acad. Sci. U. S. A..

[72] Edwards JC, Johnson MS, Taylor BL (2006). Differentiation between electron transport sensing and proton motive force sensing by the Aer and Tsr receptors for aerotaxis. Mol. Microbiol..

[73] Schweinitzer T (2008). Functional characterization and mutagenesis of the proposed behavioral sensor TlpD of *Helicobacter*
*pylori*. J. Bacteriol..

[74] Kajikawa A (2016). Characterization of flagellins isolated from a highly motile strain of *Lactobacillus*
*agilis*. BMC Microbiol..

[75] Schneider CA, Rasband WS, Eliceiri KW (2012). NIH image to ImageJ: 25 years of image analysis. Nat. Methods.

[76] Pfeiler EA, Azcarate-Peril MA, Klaenhammer TR (2007). Characterization of a novel bile-inducible operon encoding a two-component regulatory system in *Lactobacillus*
*acidophilus*. J. Bacteriol..

[77] Liong MT, Shah NP (2005). Acid and bile tolerance and cholesterol removal ability of lactobacilli strains. J. Dairy Sci..

[78] Law J (1995). A system to generate chromosomal mutations in Lactococcus lactis which allows fast analysis of targeted genes. J. Bacteriol..

[79] Altermann E (2005). Complete genome sequence of the probiotic lactic acid bacterium Lactobacillus acidophilus NCFM. Proc. Natl. Acad. Sci U. S. A..

[80] Biswas I, Gruss A, Ehrlich SD, Maguin E (1993). High-efficiency gene inactivation and replacement system for gram-positive bacteria. J. Bacteriol..

[81] Mitchell AL (2019). InterPro in 2019: Improving coverage, classification and access to protein sequence annotations. Nucleic Acids Res..

[82] Adebali O, Ortega DR, Zhulin IB (2015). CDvist: A webserver for identification and visualization of conserved domains in protein sequences. Bioinformatics.

