# Supplementary material for: Negative chemotaxis of Ligilactobacillus agilis BKN88 against gut-derived substances
Source: Sci Rep. 2023 Sep 20;13:15632. doi: 10.1038/s41598-023-42840-5 (PMC10511705; doi:10.1038/s41598-023-42840-5)
Supplement: Supplementary file 1 — Supplementary Information. [file 41598_2023_42840_MOESM1_ESM.pdf]

Supplemental Information

Negative chemotaxis of *Ligilactobacillus agilis* BKN88 against gut-derived substances

Shunya Suzuki [a,b], Kenji Yokota [a], Shizunobu Igimi [a], and Akinobu Kajikawa [a]*

[a] Department of Agricultural Chemistry, Graduate school of Tokyo University of Agriculture, 1-1-1 Sakuragaoka, Setagaya, Tokyo 156-8502, Japan.

[b] Bioproduction Research Institute, National Institute of Advanced Industrial Science and Technology, 1-1-1 Higashi, Tsukuba, Ibaraki 305-8566, Japan

***Corresponding author**
Akinobu Kajikawa, a3kajika@nodai.ac.jp

This file includes

- Supplementary Tables S1 to S4
- Supplementary Figures S1 to S7

Supplemental Tables

Table S1. Putative bile tolerance-related genes in *L. agilis* BKN88 .

**Locus tag**	**Annotation**	**Putative function**
PTL465_09640	Multidrug ABC transporter ATP-binding and permease protein	Efflux pump
PTL465_10100	Multidrug ABC transporter ATP-binding and permease protein	Efflux pump
PTL465_10110	Multidrug ABC transporter ATP-binding and permease protein	Efflux pump
PTL465_11240	Multidrug ABC transporter ATP-binding and permease protein	Efflux pump
PTL465_11250	Multidrug ABC transporter ATP-binding and permease protein	Efflux pump
PTL465_08990	Choloylglycine hydrolase	Bile salt hydrolysis
PTL465_18270	Conjugated bile salt acid hydrolase	Bile salt hydrolysis
PTL465_19440	Conjugated bile salt acid hydrolase	Bile salt hydrolysis

The putative bile tolerance-related genes were screened using NCBI database and Interpro.

Table S2. Reported chemoreceptors for Low pH, the bile constituents, and the organic acids.

**Ligand (response)[a]**	**Bacterial species**	**Receptor**	**LBD type**	**Reference**
Low pH				
High pH (+), Low pH (-)	*Escherichia coli*	Tsr	4HB	(1)
High pH (-), Low pH (+)	*Escherichia coli*	Tar	4HB	(1)
Low pH (+)	*Bacillus subtilis*	McpA	dCache_1	(2)
Low pH (+)	*Bacillus subtilis*	TlpA	dCache_1	(2)
High pH (+)	*Bacillus subtilis*	McpB	dCache_1	(2)
High pH (+)	*Bacillus subtilis*	TlpB	dCache_1	(2)
Low pH (-)	*Helicobacter pylori*	TlpA	dCache_1	(3)
High pH (+), Low pH (-)	*Helicobacter pylori*	TlpD	CZB	(3)
Bile constituents				
Sodium deoxycholate (+)	*Campylobacter jejuni*	Tlp3 (CcmL)	dCache_1	(4)
Sodium deoxycholate (+)	*Campylobacter jejuni*	Tlp4 (DocC)	dCache_1	(4)
Taurocholic acid (+)	*Vibrio cholerae*	Mlp37	dCache_1	(5)
Organic acid salts				
Lactate (+)	*Helicobacter pylori*	TlpC	dCache_1	(6)
Lactate (+)	*Pseudomonas putida*	McpP	sCache_2	(7)
Lactate (+)	*Azorhizobium caulinodans*	TlpA1	unknown	(8)
Butyrate (+)	*Pseudomonas putida*	McpS	HBM	(9)
Butyrate (+)	*Sinorhizobium meliloti*	McpV	sCache_2	(10)
Acetate (+)	*Pseudomonas putida*	McpP	sCache_2	(7)
Acetate (+)	*Pseudomonas putida*	McpS	HBM	(11)
Acetate (+)	*Sinorhizobium meliloti*	McpV	sCache_2	(10)
Acetate (+)	*Azorhizobium caulinodans*	TlpA1	unknown	(8)

[a] Attractant and repellent are shown in plus (+) or minus (-), respectively. 4HB, 4-helix bundle; dCache_1, double calcium channels and chemotaxis receptors; CZB, chemoreceptor zinc binding; sCache_2, single calcium channels and chemotaxis receptors; HBM, helical bi-modular.

Table S3. Primers used in RT-PCR.

Target gene	Primer	Sequence (5' to 3')	Product size (bp)
mcp1	DOKJ 276	ACGCGACCTAGAATCAATGG	238
	DOKJ 277	GTTTTGGCGGTTTGACTTGT	
mcp2	DOKJ 278	TAAGCAAATCAAGGGGATGC	197
	DOKJ 279	CGCTAATCCCATCAACCACT	
mcp3	DOKJ 272	GTTCAAGCTGGTGTTGCTGA	225
	DOKJ 273	AGCGCTAGCTTGCTTTTGAC	
mcp4	DOKJ 274	GCAATCGACGATGCTCACTA	156
	DOKJ 275	ACTACCGACGGCTCCCTTAT	
mcp5	DOKJ 280	GCCTACAATGTTTCCGCAAT	161
	DOKJ 281	GCGTCTTTAGGGTGAGCTTG	

Table S4. Primers used to construct MCP or CheA gene deletion mutants.

Primer	Sequence (5' to 3')	Target region
Δ*mcp1*		
DOKJ 701	ATATCTCGAGTCAAGGCAGCCTTGAAAGTG	Upstream
DOKJ 650	TTCTTCCCCCTTAGCGGCAAAAATCCTTTCTCGTA	
DOKJ 651	GAAAGGATTTTTGCCGCTAAGGGGGAAGAAGCTGT	Downstream
DOKJ 702	ATATAAGCTTATCACCTTGCGCATGAAGGCT	
DOKJ 703	CGGACTTTACAGTCGAATTTGACCG	Flanking region
DOKJ 704	GTGCCGGGCATCTTTGCAATTAAAT	
Δ*mcp2*		
DOKJ 653	ATATCTCGAGGCGCGAGGAAGAAGAAGTCG	Upstream
DOKJ 654	ATCGGCTACGTGGCCCACACTCTTGGTGCCCTTAG	
DOKJ 655	GGCACCAAGAGTGTGGGCCACGTAGCCGATTTAGC	Downstream
DOKJ 656	ATATAAGCTTGCGTGCAAGTTTCAGCAAGTC	
DOKJ 664	CGTCCAGAAGACGAAGAGTAGGAGT	Flanking region
DOKJ 665	GCCGGCTTCTTTTACCAACGGA	
Δ*mcp3*		
DOKJ 599	ATATGAATTCCGACCCCAAGACTAATCCCAACT	Upstream
DOKJ 600	TAGGCGACCGCGAACGTGCACATTAATCACAAC	
DOKJ 601	TGCACGTTCGCGGTCGCCTACTTCAAACTG	Downstream
DOKJ 602	ATATCTCGAGCTCCTGAAGAAGAGGTGCTGGAA	
DOKJ 660	CCAGGCCATCGACATGCAAC	Flanking region
DOKJ 661	GCCGCCTTGATTTCGACTCTG	
Δ*mcp4*		
DOKJ 684	ATATCTCGAGGTCAGTCTAGTGGCTACCTG	Upstream
DOKJ 685	ATGTAAGTCAGCAACACTAGGCCAACTACA	
DOKJ 686	TGTAGTTGGCCTAGTGTTGCTGACTTACAT	Downstream
DOKJ 687	ATATGGATCCACACGCTAACCTGCCAATCA	
DOKJ 692	TGTTACCGCAACTTTAAGCTCAGTTGGATA	Flanking region
DOKJ 735	TTGGGATAATTGGTAACAGCTTTGATAACC	
Δ*mcp5*		
DOKJ 688	ATATCTCGAGAAGCACCTGCTTATCTTGCA	Upstream
DOKJ 689	AGCTTCCTTAGCCACATCATTGCACATGTG	
DOKJ 690	CACATGTGCAATGATGTGGCTAAGGAAGCT	Downstream
DOKJ 691	ATATGGATCCAAGCATCGCATGGTTGGTTG	
DOKJ 694	TTGCAGCTGTAAAAAATCGGGCTCGTAAAT	Flanking region
DOKJ 734	GCAATCGCACCTAAGGCCATATCATCATTG	
Δ*cheA*		
DOKJ 1312	ATATCTCGAGCATCATCCTAACCGGGATGG	Upstream
DOKJ 1313	TAGCTATTCGCCACCAGCCATTATTTTTCCTCCTC	
DOKJ 1314	GGAAAAATAATGGCTGGTGGCGAATAGCTAGTGGC	Downstream
DOKJ 1422	ATATGGATCCCCCAAAATTAGGAAAGGGCC	
DOKJ 1316	TCGGCGGCTGCTTACTACGG	Flanking region
DOKJ 1317	TACTTCTTCAACGTCGTCTGCAGAG	

Supplemental Figures

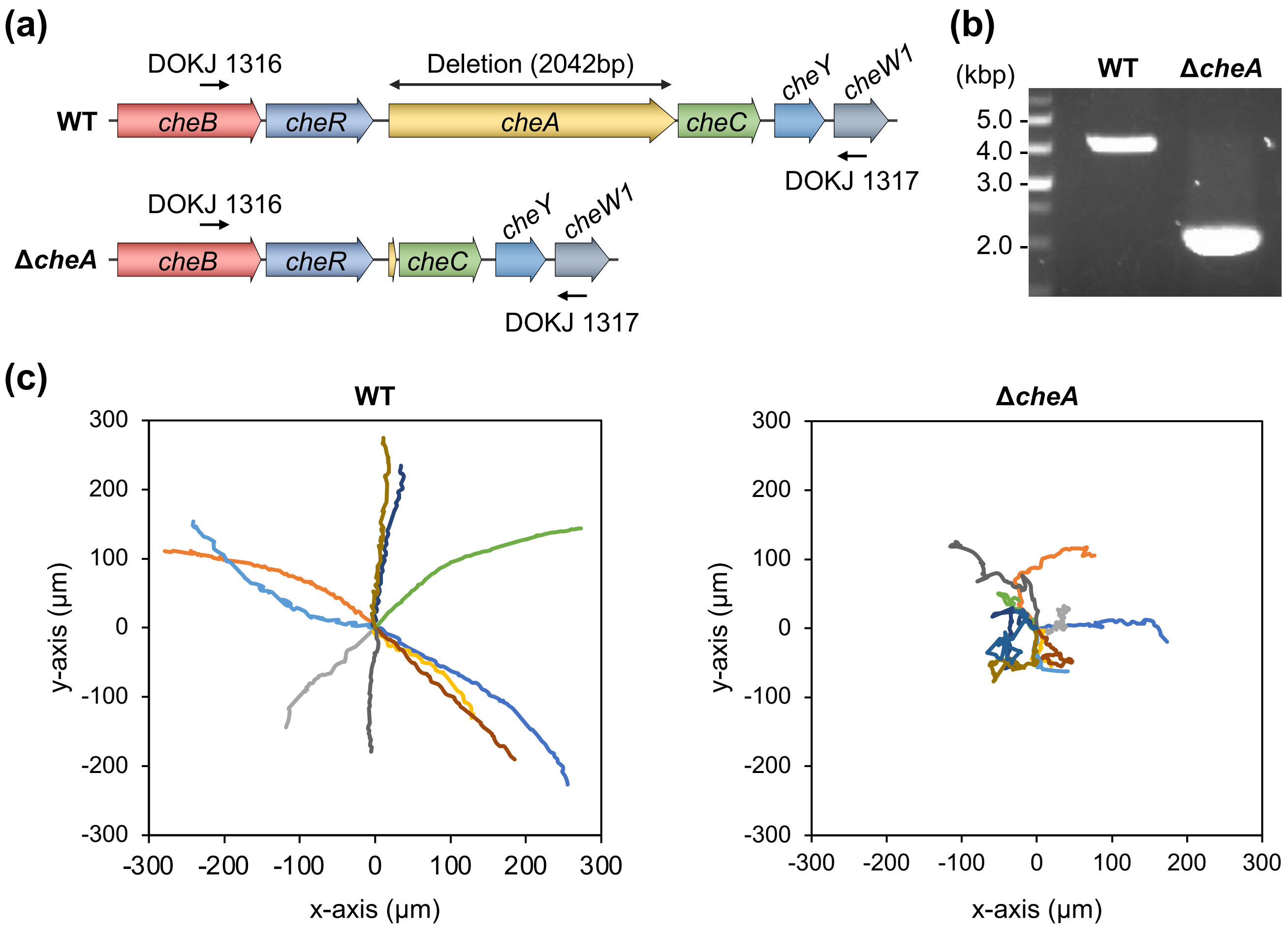

Figure S1 Construction of a non-chemotactic mutant (Δ*cheA*) of *L. agilis*. (a) Partial genetic map of the chemotaxis-related genes in the wild-type (WT) and Δ*cheA* strains of *L. agilis*. PCR amplification primers to confirm the CheA gene deletion are shown in the arrows with primer names. (b) Validation of the CheA gene deletion by PCR using the primers. (c) Analysis of swimming behaviors of the WT and Δ*cheA* strains. Individual motile cells (n=10) were tracked for 10s (15 flames per second) by using a BZ-X710 microscope (KEYENCE) with the video editing analysis software, VW-H2MA (KEYENCE). All coordinates (x,y) of the starting point of each tracking were set to (0,0).

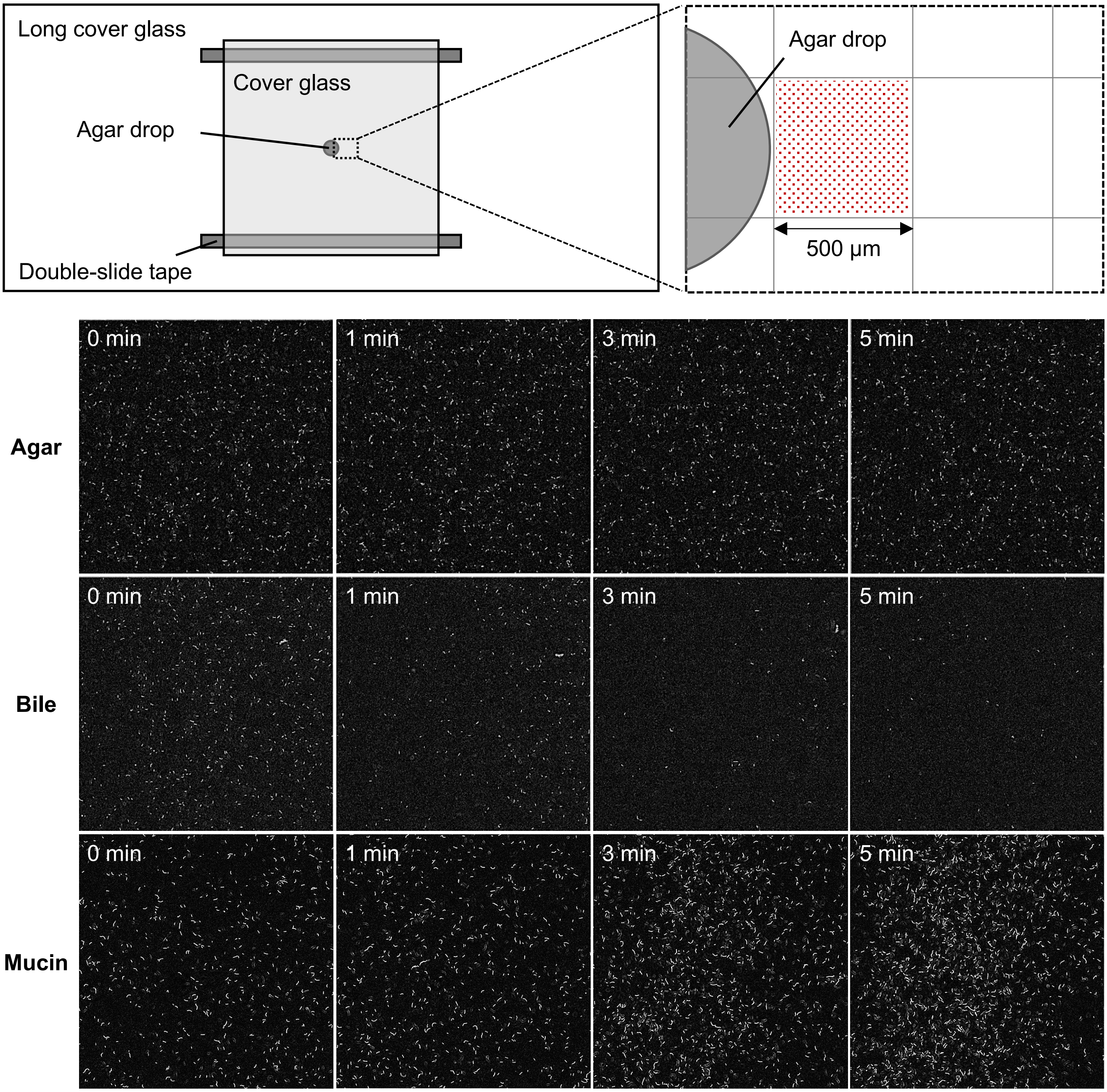

Figure S2 Microscopic agar-drop assay. Microscopic agar-drop assay was performed according to the method of Islarm et al. (12) with minor modifications. Upper: Schematic representation of the flow chamber used for the microscopic agar-drop assay. Test reagent in distilled water containing 1.5 % (wt/vol) agar was dropped onto the center of a long cover glass, and then a cover glass was attached to the long cover glass using double-sided tape. Bacterial cells in exponential phase were harvested and suspended in MRS medium. The bacterial suspension was diluted to 1:15 with chemotaxis buffer and infused into the flow chamber. The motile cells near the agar drop were immediately observed and recorded using a time-lapse microscope every 1 min. The number of cells in the red dotted area was counted using ImageJ software. Lower: Representative microscopic images around the agar drop (the red dotted area) containing bile or mucin. An agar drop without test chemicals was used as a control.

F_1F_0-ATPase operon in *L. agilis* BKN88

BLAS01000074.1 (91,500:98,100)

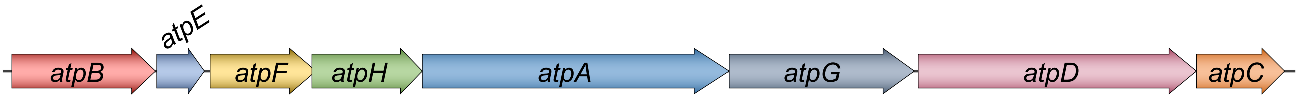

Figure S3 A genetic map of F_1F_0-ATPase operon in *L. agilis* BKN88 genome. The genome accession number and the position are shown in figure.

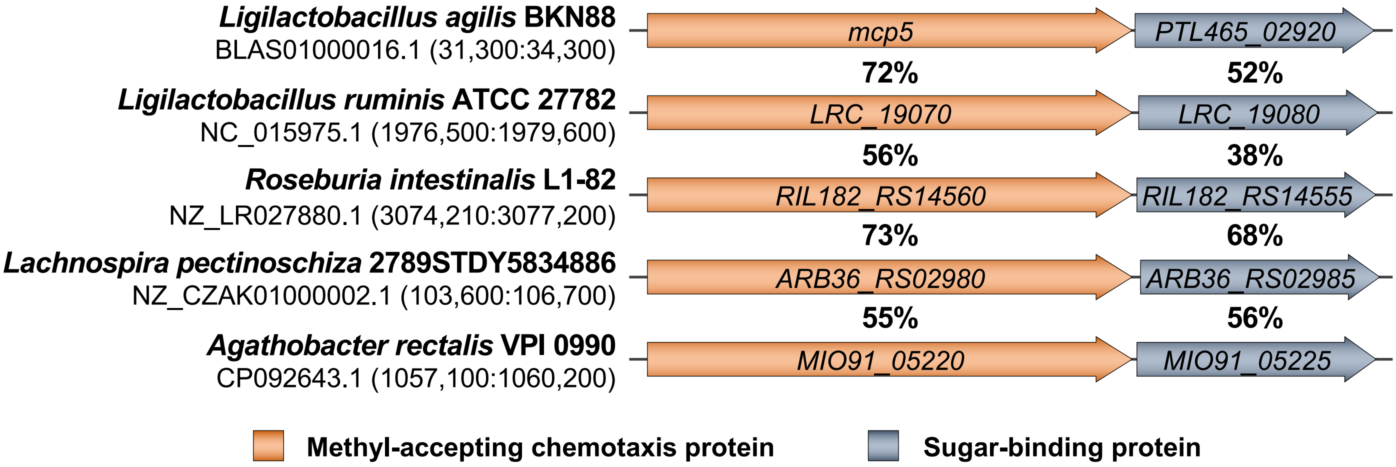

Figure S4 Comparison of Mcp5 gene cluster in *L. agilis* BKN88 and human gut-derived commensal bacteria. Genome accession numbers and positions are shown on left. The locus tag numbers are indicated inside the arrows. The amino acid sequence of each methyl-accepting chemotaxis protein and sugar-binding protein were analyzed by multiple alignments using ClustalW, and the amino acid identity (%) between the proteins is shown.

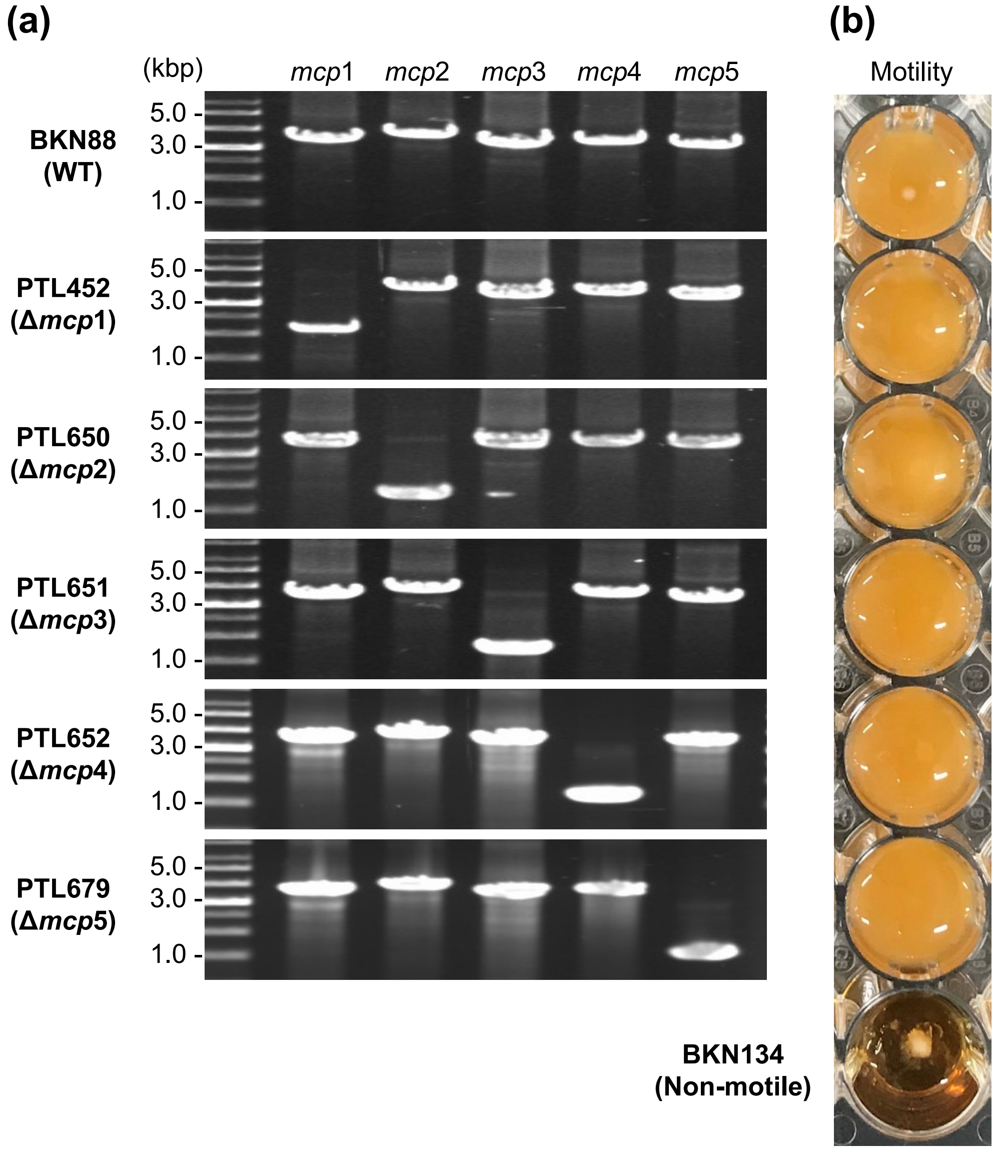

Figure S5 Construction of the MCP gene deletion mutants. (a) Validation of the MCP gene deletions by PCR. (b) The motility of the MCP gene deletion mutants. The motility of the constructed mutants was observed with cultivation in semi-solid MRS medium.

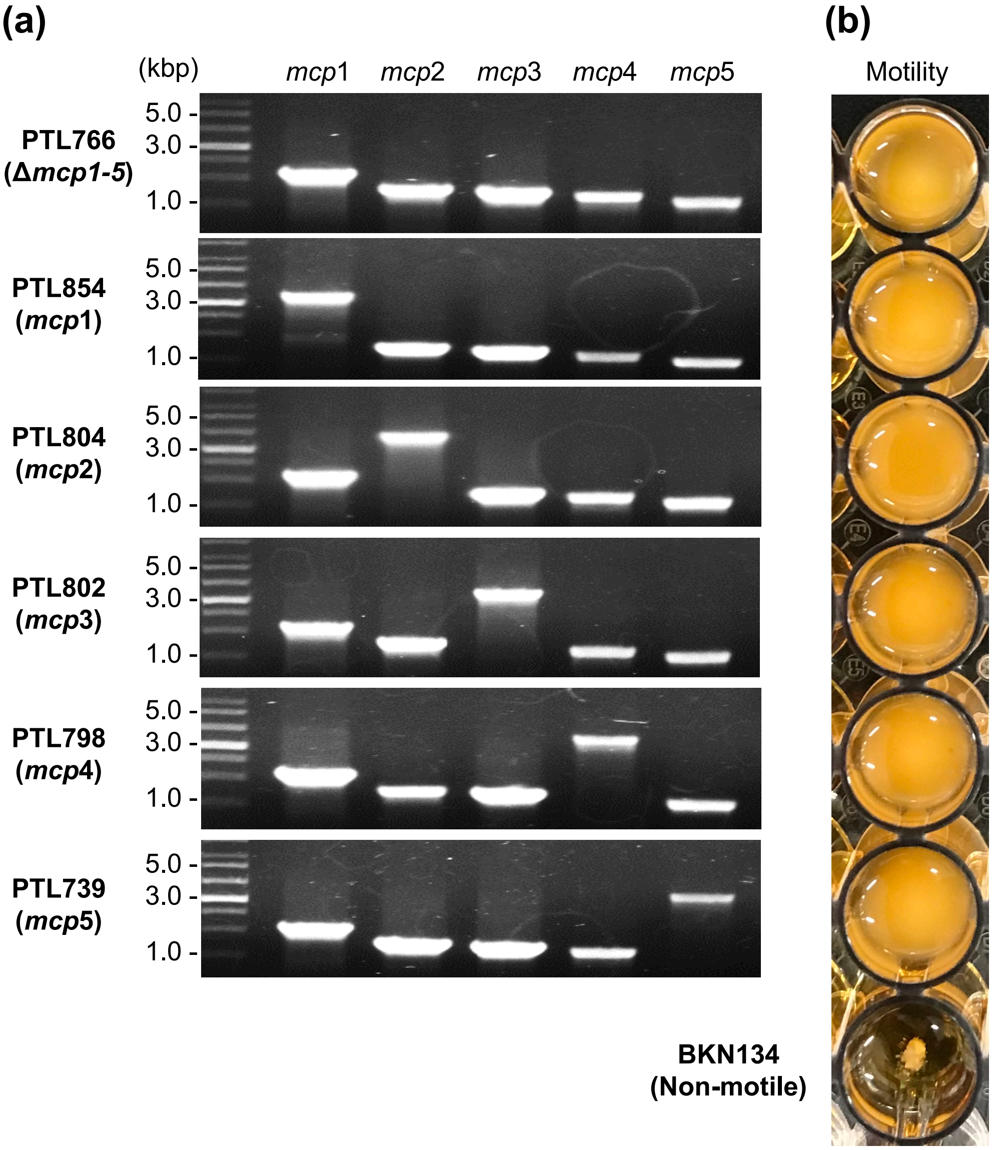

Figure S6 Construction of *L. agilis* mutants expressing each of the MCP genes. (a) Validation of the MCP gene deletions by PCR. (b) The motility of the MCP gene deletion mutants. The motility of the constructed mutants was observed with cultivation in semi-solid MRS medium.

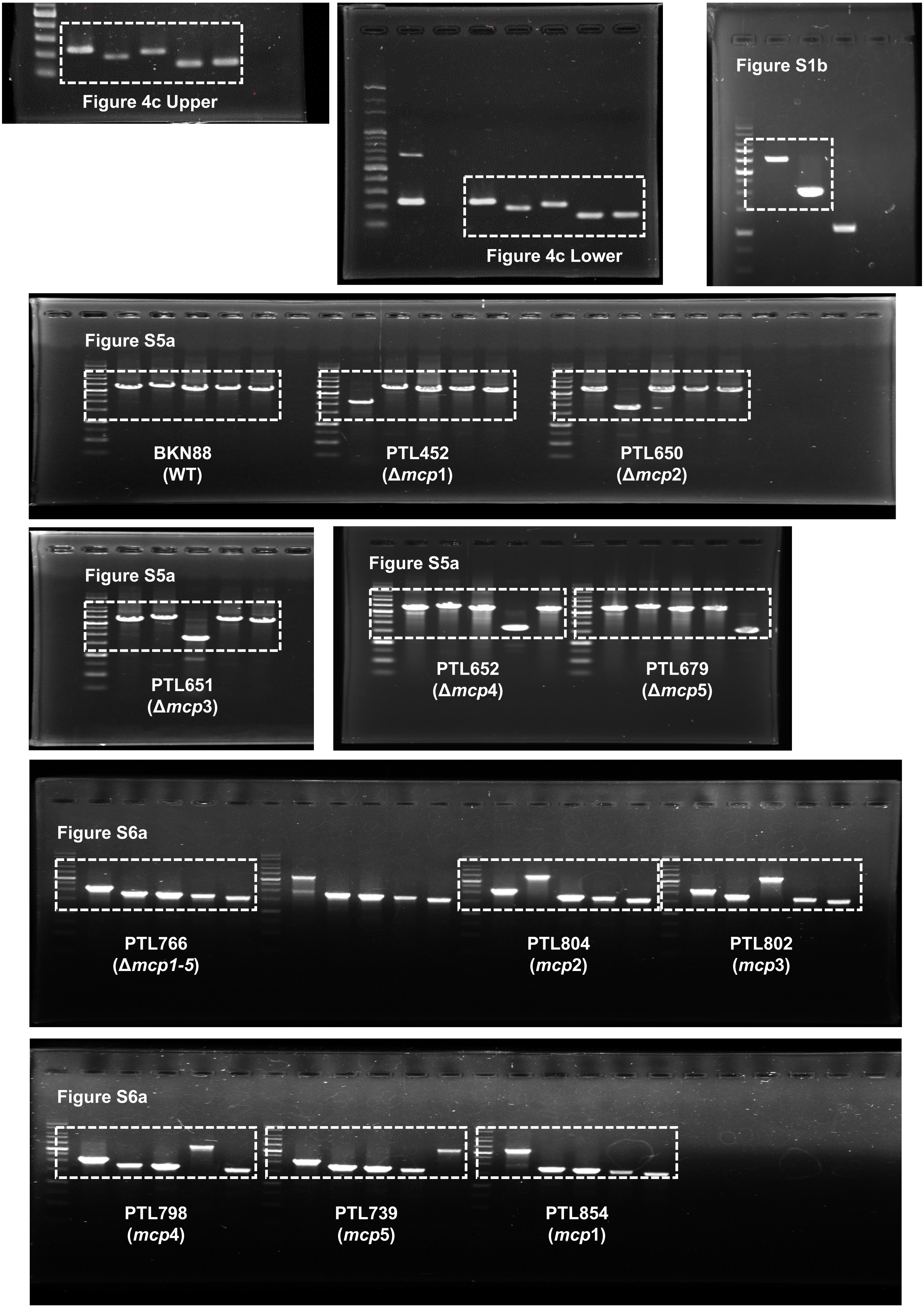

Figure S7 Original images for cropped data in Figure 4c, S1b, S5a, and S6a. Dashed squares correspond to the bands showed in the figures.

References

1. Yang Y, Sourjik V. *Mol Microbiol*. 2012 Dec;86(6):1482-9.
2. Tohidifar P *et al*. *J Bacteriol*. 2020 Jan 29;202(4):e00491-19.
3. Huang JY *et al*. *PLoS Pathog*. 2017 Jan 19;13(1):e1006118.
4. Li Z *et al*. *J Med Microbiol*. 2014 Mar;63(Pt 3):343-354.
5. Nishiyama S *et al*. *Sci Rep*. 2016 Feb 16;6:20866.
6. Machuca MA *et al*. *Sci Rep*. 2017 Oct 26;7(1):14089.
7. García V *et a*l. *Appl Environ Microbiol*. 2015 Aug 15;81(16):5449-57.
8. Liu X *et al*. *Front Microbiol*. 2019 Dec 3;10:2727.
9. Lacal J *et a*l. *J Biol Chem*. 2010 Jul 23;285(30):23126-36.
10. Compton KK *et al*. *J Bacteriol.* 2018 Nov 6;200(23):e00519-18.
11. Pineda-Molina E *et al*. *Proc Natl Acad Sci U S A*. 2012 Nov 13;109(46):18926-31.
12. Islam MS *et al*. *FEMS Microbiol Lett*. 2014 Jul;356(1):39-44.
